# Recent Developments in Cellulose/Chitosan Biopolymer Composites for Winery Wastewater Treatment and Reuse: A Review

**DOI:** 10.3390/ma18215028

**Published:** 2025-11-04

**Authors:** Fisokuhle Innocentia Kumalo, Innocent Mugudamani, Ernestine Atangana, Thandi Patricia Gumede

**Affiliations:** 1 Department of Life Sciences, Faculty of Health and Environmental Sciences, Central University of Technology, Bloemfontein 9301, Free State, South Africa; kumalofisokuhle@gmail.com; 2 Department of Civil Engineering, Centre for Sustainable Smart Cities, Faculty of Engineering, Built Environment and Information Technology, Central University of Technology, Bloemfontein 9301, Free State, South Africa; imugudamani@gmail.com; 3 Centre for Environmental Management, Faculty of Natural and Agricultural Science, University of Free State, Bloemfontein 9301, Free State, South Africa; atanganae@ufs.ac.za

**Keywords:** winery wastewater, cellulose/chitosan composites, polyvinyl alcohol, polyethylene glycol, polyacrylamide, flocculation, adsorption, irrigation reuse

## Abstract

Winery wastewater, characterized by high organic load, fluctuating pH, and seasonal variability, presents a major environmental challenge for sustainable water management in viticulture regions. Recent advances in bio-based polymer composites, particularly those incorporating cellulose and chitosan matrices blended with synthetic polymers such as polyacrylamide (PAM), polyvinyl alcohol (PVA), and polyethylene glycol (PEG), provide promising possibilities for effective wastewater treatment and water reuse in irrigation. This review critically explores the synthesis, structural properties, and functional performance of cellulose/chitosan-based composites, with a particular emphasis on their adsorption, flocculation, and biodegradability in the context of winery effluent treatment. Evidence from recent laboratory- and pilot-scale studies highlights the significance of pH-responsive functional groups, electrostatic interactions, and hydrogen bonding in controlling pollutant capture and regeneration efficiency. While notable removal efficiencies of these composites have been demonstrated to exceed 85–95% for COD, 80–98% for turbidity, and >90% for heavy metals, challenges remain in terms of regeneration, long-term field applicability, and scale-up. Overall, biopolymer composites represent a promising pathway toward sustainable wastewater treatment and irrigation reuse in winery operations.

## 1. Introduction

In recent years, freshwater has become a crucial resource in South Africa due to high levels of pollution in aquatic environments. Wine-making industries are a notable source of effluent loading [1,2]. The number of wineries in South Africa has increased from less than 300 in 1997 to about 650 in 2020, producing winery wastewater (WWW) of approximately 980,000 m^3^ annually [3]. Winery wastewater is the effluent generated during the various stages of wine production. These stages include crushing and pressing of grapes, cleaning tanks, washing floors and equipment, rinsing transfer lines, cleaning barrels, managing wine and product losses, bottling, and operating filtration units [4]. This wastewater is compositionally complex and seasonally variable. It typically exhibits high chemical oxygen demand (COD), total suspended solids (TSS), low pH, heavy metals, organic compounds, and varying salinity and nutrient levels. In WWW, pH commonly ranges from 3 to 6, while COD and BOD frequently fall in the (COD: 2–20 g/L; BOD: 1–10 g/L) ranges; phenolic compounds and colour are also significant contributors to toxicity. If discharged untreated, winery wastewater can cause major environmental impacts [5]. This increased demand for wine has increased pressure on soil, vegetation, and water resources [6].

The environmental impact caused by the release of WWW without proper treatment can be problematic for ecosystems, due to the contamination of water. This may include oxygen depletion and eutrophication in rivers, wetlands, and natural streams. This is due to the fast consumption of dissolved oxygen, which reduces oxygen availability for aquatic and amphibious life [7,8]. It may further lead to air pollution which may be caused by foul odours released from volatile of organic compounds (VOCs), and the potential for the formation of secondary pollutants. These odours, if not managed properly, can impact air quality [9]. In addition, excessive sodium and salinity in treated or untreated WWW effluent can impair soil structure, reduce infiltration, and negatively affect plant growth. Similar challenges have been documented in Mediterranean wine-growing regions, Australia, and California, highlighting the global relevance of this problem [8,10,11]. Previous research studies have focused on general wastewater, yet few studies have synthesized winery-specific performance of cellulose/chitosan composite blended with PAM, PVA, and PEG according to adsorption, flocculation, and membrane map removal irrigation criteria (for PH, EC, SAR, metals). This review addresses this gap by pairing mechanisms with reuse threshold and deployment constraints.

Conventional treatment technologies including activated sludge, membrane filtration, and chemical coagulation have been widely applied. However, they often require high energy and cost and may generate secondary pollutants. In response to the limitations, bio-based polymer composites have emerged as eco-friendly, low-cost, and efficient alternatives for wastewater treatment. Among these, cellulose and chitosan have received substantial attention due to their excellent biodegradability, hydrophilicity, and ability to form functional composites with enhanced adsorption and flocculation capabilities [12]. Beyond adsorption/flocculation, these matrices can be fabricated into membranes, beads, aerogels, and hydrogels that provide high surface area and adjustable porosity for WWW remediation. However, these biopolymers may exhibit limitations such as poor film-forming ability for cellulose and susceptibility to swelling for chitosan [13]. To address these challenges, the development of biopolymer composites using polymer matrices such as polyacrylamide (PAM), polyvinyl alcohol (PVA), and polyethylene glycol (PEG) blended with cellulose/chitosan combines their complementary properties to improve mechanical stability, processability, and pollutant removal. PAM, PVA, and PEG have received attention in the treatment of wastewater due to their distinctive properties such as biodegradability, and biocompatibility [14,15,16]. When appropriately formulated, these polymers can act as flocculants, support matrices, or modifiers that enhance adsorption kinetics, stability under varying pH/ionic strength, and reusability.

This review therefore aims to provide a comprehensive analysis of recent progress in cellulose- and chitosan-based polymer composites modified with PAM, PVA, and PEG for winery wastewater treatment. It evaluates how surface modification, polymer blending, and composite preparation have enhanced adsorption and flocculation efficiency. Unlike previous reviews that broadly discuss wastewater remediation, this work uniquely contextualizes findings for winery effluents, integrates South African and international case studies, and emphasizes polymer matrix composites as scalable, sustainable technologies. Furthermore, this work identifies existing research gaps related to scale-up feasibility, techno-economic assessment, and compliance with irrigation reuse standards, thus providing possible alternatives for sustainable and resource efficient winery wastewater management. It is structured as follows: Section 1 introduces the context, environment implications, conventional technologies, and proposed sustainable technologies. Section 2 describes the key physicochemical characteristics of winery wastewater. Section 3 reviews current treatment technologies and their limitations. Section 4 reviews the effectiveness of bio-based polymer composites for water and WWW treatment. Section 5 focuses on the integration of cellulose and chitosan with polymer matrices (PAM, PVA, PEG) for wastewater applications. Section 6 outlines synthesis routes and characterization methods for the composites. Section 7 discusses their reuse potential for irrigation and reviews relevant South African water quality standards. Section 8 concludes with a summary of key findings, mechanisms, and future directions for improving sustainability in wastewater reuse.

## 2. Characteristics of Winery Wastewater

### 2.1. Sources and Generation

**Figure 1:**
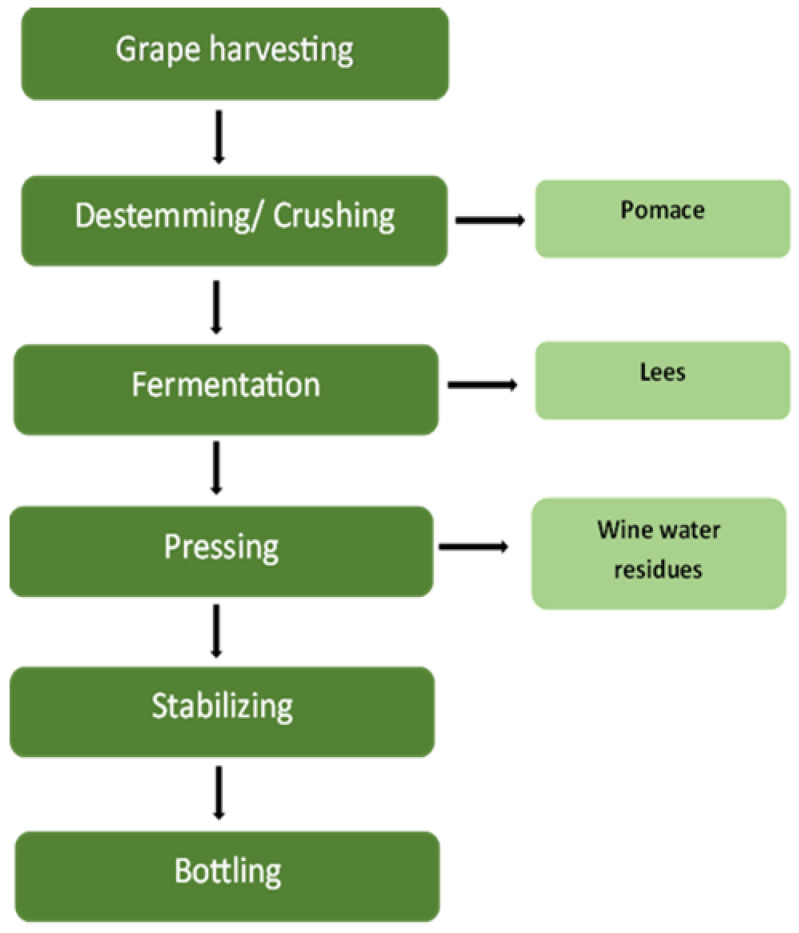
Wine-making process with generated residues.

Winery wastewater (WWW) is generated at several phases of the wine-making process, with each phase providing distinct physical and chemical qualities that influence treatment complexity and environmental implications. The key sources of WWW are crushing, fermentation, pressing, stabilization, and equipment cleaning (Figure 1) [4]. During the crushing stage, large quantities of suspended solids, sugars, and polyphenolic compounds from grape skins and pulp are released into the effluent [5]. These organic constituents result in elevated biochemical oxygen demand (BOD) and chemical oxygen demand (COD) levels, posing a risk of oxygen depletion in receiving waters if not adequately treated [6]. Yang et al. [17] estimated that approximately 20–30% of grape pomace is discarded after crushing, contributing significantly to the organic load. The fermentation process, which is a critical step in converting carbohydrates into alcohols or acids, produces wastewater including ethanol, volatile fatty acids, and yeast biomass, all of which increase organic load and decrease pH. Maicas and Mateo [18] observed that different microbial species are used during fermentation to improve wine quality, but their metabolic by-products complicate wastewater composition. The acidic and high-organic effluent from fermentation can upset microbial equilibrium in treatment systems, needing neutralization or dilution prior to biological processing [19]. During pressing and stabilization, large amounts of turbid effluent containing phenolic compounds are created. Subsequent operations, such as racking, fining, and filtration, add pollutants such as bentonite, sulfites, and tartrates, which can be hazardous to aquatic creatures and impede microbial activity in biological treatment systems [18]. As a result, chemical monitoring and correction are essential before discharge or reuse. The cleaning and sanitation phase generates the majority of wastewater, which frequently contains spilled wine, detergents, acids, and alkalis, leading to pH fluctuations and increased total dissolved solids (TDSs) [19]. According to Mosse et al. [5], this step can produce up to 80% of total winery effluent, making it an important target for volume reduction and pH management. Understanding how each production stage influences the physicochemical properties of winery wastewater is critical for developing stage-specific management and treatment techniques that meet environmental discharge laws. Wastewater generation usually peaks during the vintage season [18,19,20]. Figure 1 depicts the vinification process and the resulting leftovers, which include winery wastewater.

### 2.2. Physicochemical Properties of Winery Wastewater

Winery wastewater has several unique chemical and physical characteristics that can have an impact on the environment if not managed properly. These may include high organic load, high chemical oxygen demand (COD), biological oxygen demand (BOD), low pH, colour, turbidity, temperature, salinity, solid content (suspended and total dissolved solids), and odour [19]. Further WWW may contain high levels of concentrations of potassium (K^+^) and sodium (Na^+^), sugars, ethanol, glycerol, esters, organic acids, and phenols from grapes and wine, as well as cleaning agents. Although all these parameters may be used to evaluate winery wastewater, COD, BOD, pH, SAR, EC, K^+^ and Na^+^ are considered to be important [20]. Table 1 illustrates the typical ranges of WWW effluents from different literature.

#### 2.2.1. High Organic Load

Winery wastewater may contain a variety of organic loads, which may include a high content of sugar, which is generated from the crushing of grapes. This is usually the most contributing factor to organic load. Ethanol from the fermentation process and other organic contaminants, such as glycerol, esters, and phenols from the cleaning agents, also contribute to the high level of organic loads in WWW. These may further contribute to the prevalence of nutrients such as nitrogen and phosphorus in winery wastewater. Their environmental effects include foul odours and an excess of nutrients in the wastewater [9].

#### 2.2.2. Chemical Oxygen Demand (COD)

Chemical oxygen demand (COD) measures the amount of oxygen needed to chemically oxidize organic matter in the water. WWW is characterized by high levels of COD which is caused by the presence of organic compounds such as sugars, ethanol, and organic acids [24,29]. The COD concentration of winery effluents ranges from 320 to 49,105 mg/L. Winery wastewater with high levels of chemical oxygen demand (COD) may have a number of adverse effects such as eutrophication, toxicity, water contamination, and soil degradation on the environment and irrigation systems [24,30]. High COD levels in water bodies limits the dissolved oxygen (DO), which results in the suffocation of aquatic life [5]. When water with high COD is used for irrigation, its decreases the microbial activities in the soil causing depletion of oxygen, anaerobic conditions, and further release of odorous compounds which may further inhibit plant growth. Although moderate COD levels can improve soil fertility by contributing organic matter and microbial food sources, excessive COD (>400 mg/L) can cause soil clogging, reduced infiltration, and phytotoxicity. Therefore, COD must be balanced [25,31].

#### 2.2.3. Biological Oxygen Demand (BOD)

The biological oxygen demand (BOD) refers to the amount of dissolved oxygen (DO) consumed by biological organisms when they decompose organic matter in water. High BOD indicates significant pollution, as it leads to oxygen depletion in the water, potentially resulting in harming aquatic life and causing unpleasant odour [32]. BOD values in winery wastewater tend to be around 60.5 to 66% of the COD [24]. Water with high BOD may also cause oxygen depletion in the soil when used for irrigation, causing further eutrophication. Hence, BOD must be maintained.

#### 2.2.4. pH Effects

The presence of organic acids such as malic and citric acid in WWW typically reduces the pH of water to about 3 to 5, resulting in WWW being more acidic. This acidity reduces the availability of nutrients such as phosphorous and calcium in the soil, which further results in reduced plant growth [7,33]. Cleaning agents that contain a high concentration of sodium bicarbonate often cause an increase in pH in WWW. The pH further affects the stability of microbes, thus maintaining it between 6.0 and 9.0 can minimize the potential for negative impacts to soil biological treatment, crop growth, and groundwater quality [34].

#### 2.2.5. Sodium Adsorption Ratio (SAR)

The disposal of WWW can negatively affect the environment since WWW contains a high concentration of potassium (K) due to the presence of acid grape juice and high concentration of sodium (Na), which results from alkaline cleaners used for sanitation. The accumulation of high concentrations of K and Na in the environment has been shown to change soil structure by increasing clay dispersion and reducing the distribution of mesopores and micropores. This ultimately leads to a reduction in plant growth and reduction in soil infiltration rates, which further result in surface runoff and soil erosion [4,10,32]. It further causes soil dispersion and aggregation, leading to changes in soil structure and water infiltration. Salinity levels also tend to increase the SAR level, which can result in high concentrations of sodium (Na^+^) and potassium (K^+^) [35,36]. High sodium concentrations in winery wastewater can cause osmotic stress in grapevines, impacting water uptake and overall growth. Accumulation of K^+^ in soil could lead to soil structure instability in the long term [37]. While short-term irrigation can improve soil cation exchange and microbial activity, long-term use with high SAR causes irreversible soil deterioration. Therefore, dilution, mixing, or treatment may be required before reuse.

### 2.3. Environmental and Health Concerns

Winery wastewater may have both environmental and health risks due to its high organic load (sugars, acids, and alcohol), as well as the presence of pesticides and other chemicals used during wine-making [38]. Winery wastewater can cause the salinization and eutrophication of water resources such as natural streams, rivers, dams, and groundwater. Furthermore, wastewaters can cause soil solidity, salinity, contamination with a wide range of chemicals, waterlogging, and anaerobiosis, as well as loss of soil structure and increased susceptibility to erosion. Where solid wastes are present, offensive odours may be generated and may result in the contamination of soil and water resources, inhibiting the vegetative performance [24,38]. Wastewater can contain pathogens and chemicals, including pesticides, that can be harmful to humans from exposure or if ingested, which can further cause various illnesses, potentially impacting human health if not treated properly. Prolonged exposure to contaminated water or soil can also lead to various health problems, including respiratory issues and skin irritation [39,40,41,42]. In response to these environmental challenges, several treatment techniques have been employed to address these issues caused by disposal of WWW into water bodies.

## 3. Treatment Techniques for Winery Wastewater

### 3.1. Overview of Conventional Methods

There is wide range of methods used for the treatment of WWW and those include biological, physicochemical, membrane filtration and separation, and advanced oxidation processes (AOPs) [30].

#### 3.1.1. Biological Treatment of Winery Wastewater

Several systems are currently offered by technology providers and current research envisages the availability of new promising technologies for winery wastewater treatment. The treatment of winery wastewater can be achieved using several biological processes based both on aerobic or anaerobic systems, using biomass or biofilms [43]. Aerobic processes utilize the microorganisms that consume organic matter in the presence of oxygen. While anaerobic processes occur in the absence of oxygen whereby the anaerobic bacteria convert organic matter into carbon dioxide and methane [2,44].

Biological treatment methods have been widely studied and applied for the remediation of winery wastewater due to their effectiveness in reducing organic pollutants. The literature presented in Table 2 highlights both aerobic and anaerobic biological processes as key approaches, each with distinct advantages and challenges. Aerobic treatment systems such as air-microbubble bioreactors (AMBBs) have demonstrated high removal efficiencies of (COD) in winery wastewater. For instance, after extended treatment, AMBBs can achieve about 99% removal of COD [45]. This is due to enhanced oxygen transfer through specialized aeration techniques like Venturi injectors. This aligns with the broader literature indicating that sufficient oxygen supply is crucial for maximizing microbial degradation of organic compounds in aerobic systems [46]. The ability of AMBBs to operate in both batch and continuous modes adds operational flexibility, making them suitable for varying wastewater volumes common in seasonal winery operations. Similarly, the jet-loop activated sludge reactors (JLRs) have also been recognized due to their efficient COD removal, which often exceeds 90% as reported by [47]. The high turbulence and mixing rates of JLR designs optimize oxygen transfer and contact between microbes and pollutants, consistent with findings in other industrial wastewater treatments [48]. Their relatively low energy requirements make them attractive for wineries with limited space or energy constraints.

Despite these benefits, aerobic biological systems face challenges such as membrane fouling and high operational costs [49]. Moreover, anaerobic processes provide an alternative biological treatment approach with notable economic and environmental advantages. For instance, the expanded granular sludge bed (EGSB) reactor has been shown to effectively remove up to 96% of COD [50], effectively reducing pollutant loads below discharge limits. This is due to efficient mass transfer and biomass retention, producing methane-rich biogas that can be harnessed as a renewable energy source [51]. Furthermore, anaerobic sequencing batch reactors (ASBRs) have also shown particular promise for phenol removal, achieving nearly complete (100%) degradation within short retention times [52]. Their flexibility, allowing different configurations and phases, is well documented in studies on industrial wastewater treatment [53]. Nonetheless, ASBRs may require supplementary treatment to address pathogen removal and are typically constrained by lower organic loading capacities compared to continuous-flow systems.

Overall, the literature shows that biological treatments are effective for winery wastewater, especially in reducing effluents such as COD and phenolic compounds. Aerobic systems provide faster treatment and higher pathogen removal but at higher operational costs and energy demand, while anaerobic processes are more energy-efficient and yield valuable biogas but can be more sensitive to operational fluctuations and may need complementary treatment steps. Therefore, future research in the literature suggests integrating biological treatments with physicochemical or advanced oxidation processes to enhance overall efficiency.

**Table 2:** Performances, advantages, and drawbacks of biological treatments of winery wastewater.

Biological Treatment Used	Main Findings	Advantages	Limitations	References
Aerobic biological treatment				
Air-microbubble bioreactor (AMBB)	The COD wastewater ranged between 4.0 and 8.0 kgCODm^−3^ and the efficiency of the batch treatment was about 90.0 ± 4.3%, after 6 days of operation. The maximum efficiency (99%) was obtained after 15 days of treatment.	It uses a Venturi injector in conjunction with mass transfer multiplier nozzles, which allow an efficient oxygen transfer. It can also operate in batch or continuous conditions.	High cost and membrane fouling.	[45]
Jet-loop activated sludge reactor (JLR)	The COD removal efficiency was higher than 90% with an organic load of the final effluents between 0.11 and 0.3 kg-COD m^−3^.	The JLR produces high mixing and turbulence that ensures optimal mass transfer and good biological conversion. It is also characterized by reduced volumes, which means limited area requirements, reduced costs, and limited energy consumption.	May form sludge with poor settling characteristics and rapid membrane fouling.	[47]
Anaerobic biological treatment				
Hybrid (Expanded Granular Sludge Bed) EGSB reactor	COD removal efficiency reached 96% where COD concentration was reduced below the maximum limit for discharge to a water body.	Mass transferring with separators allowing collection and recirculation of the gas and liquid streams.	Possibility for sludge washout and sensitivity to influent variations.	[50]
Anaerobic Sequencing Batch Reactors (ASBRs)	The one-stage reactor used for removal of phenol presented better results for the removal of phenol, reaching 100% removal in a period of 10 days at a concentration of 210 mg/L. Whereas the two-stage near 100% removal, the methanogenic reactor, presented greater degradation of the remaining phenol.	It allows great flexibility of operation as it can work in different modalities. It also allows the control of the intermediate products.	Lower ability to remove pathogen in wastewater treatment systems. Lower organic loading capacity and possibility to require more than one unit in some cases.	[52]

#### 3.1.2. Membrane Filtration and Separation Technique for Treatment of Winery Wastewater

Membrane filtration and separation techniques are some of the effective methods for treating winery wastewater. These techniques include microfiltration (MF), ultrafiltration (UF), nanofiltration (NF), and reverse osmosis (RO). MF is primarily used for removing suspended solids, colloids, and some bacteria, while UF is mostly used to remove larger molecules like tannins, proteins, and some polysaccharides. NF and RO are commonly used to remove dissolved salts, small organic molecules, and other pollutants. Different studies [54,55] have been conducted to study the effectiveness of these techniques.

Granados et al. [54] investigated the different membrane separation processes like microfiltration (MF), ultrafiltration (UF), nanofiltration (NF), and reverse osmosis (RO) for the recovery of polyphenols from winery wastewater. Their findings revealed that MF and UF membranes removed suspended solids and colloids from the extracts. NF was useful for polyphenols separation and RO membranes were able to concentrate polyphenol streams of 86% from lees filters. Furthermore, Bottino et al. [55] also studied the treatment of olive mill wastewater through integrated pressure-driven membrane processes. The results demonstrated that the RO technique rejected more than 99.3% of phenolic compounds and produced water with low salinity, chemical oxygen demand, and phytotoxicity.

Weng et al. [56] also reported on the development of ultrafiltration and nanofiltration using cellulose composites. In their findings, they reported that both ultrafiltration and nanofiltration membranes had the capacity to absorb about 75% and 88.6% of COD and ammonium in wastewater, respectively. Although the results from these previously conducted studies revealed that membrane filtration may be effective in removing phenols in the liquids, there is still not enough capability in removing other pollutants such as metal ions in the wastewater. This may be due to different experimental conditions such as high pressure which may further cause the defouling of membranes.

#### 3.1.3. Physicochemical Methods for Treatment of Winery Wastewater

Physicochemical processes for the treatment of WWW may include sedimentation with the addition of flocculants, chemical precipitation with chelating agents, coagulation/flocculation, and electrocoagulation (EC). These methods have gained considerable attention for winery wastewater management due to their effectiveness in removing suspended solids, organic matter, and other pollutants. Table 3 shows previous studies conducted for WWW treatment using various physicochemical processes [30].

The use of natural coagulants like chitosan has been demonstrated as an eco-friendly and effective approach for reducing key water quality parameters such as COD, TSS, and turbidity. A comparative study by Braz et al. [57], involving four chemical coagulants—ferric sulfate (FeSO_4_), aluminium sulfate (Al_2_(SO4)_3_), ferric chloride (FeCl_3_), and calcium hydroxide (Ca(OH)_2_)—revealed high removal efficiencies across multiple parameters. COD removal reached 84.5%, while turbidity and TSS removal were even higher, at 96.6% and 99.1%, respectively. Volatile suspended solids (VSSs) were also effectively reduced by 98.7%. These findings align with the well-established use of metal salt coagulants in wastewater treatment, where hydrolysis products destabilize colloidal particles and promote aggregation [58]. Among the coagulants tested, FeCl_3_ often shows superior performance in terms of turbidity removal, attributed to its strong charge neutralization and sweep flocculation capabilities. According to Rizzo et al. [59], chitosan obtains about 80% removal of TSS, 92% reduction in turbidity, and 73% decrease in COD in winery wastewater. These results are consistent with other studies highlighting chitosan’s biocompatibility, biodegradability, and strong adsorption capacity for winery effluents [60]. Chitosan’s cationic nature facilitates charge neutralization of negatively charged particles, promoting effective coagulation. Another study summarized in the table below [61] documented coagulation/flocculation achieving 95% turbidity reduction, 97% TSS removal, and 54% COD reduction.

Electrocoagulation (EC) uses electrical currents to generate coagulant species in situ by dissolving sacrificial anodes, typically iron or aluminium. As reported in the study by Kirzhner et al. [62], EC treatment of winery wastewater achieved removal rates of 16.4–27.9% for biochemical BOD, 28.2–41.9% for COD, and an impressive 89.2% for total phosphorus (TP). While the removal efficiencies for organic loads were moderate compared to chemical coagulation, the high phosphorus removal underscores EC’s potential in nutrient control, which is vital for preventing eutrophication when treated water is discharged or reused. EC also offers operational advantages such as reduced chemical usage and sludge volume and easier automation [63]. However, energy consumption and electrode maintenance remain challenges for widespread adoption.

Collectively, the literature supports that coagulation and flocculation, whether using natural polymers or conventional metal salts, effectively reduce suspended solids and turbidity in winery wastewater, but may be less effective for dissolved organic pollutants like COD. Electrocoagulation offers promising nutrient removal, especially for phosphorus, which is less addressed by traditional coagulants. Future research trends emphasize combining physicochemical methods with biological treatments and exploring sustainable, low-cost coagulants to optimize treatment efficiency and economic feasibility in winery wastewater management.

**Table 3:** Previous studies conducted for physicochemical treatment in winery wastewater.

Flocculant/Coagulant	Method of Treatment	VSS (%)	TSS (%)	COD (%)	Turbidity (%)	Nutrients	References
FeSO_4_, Al_2_(SO_4_)_3_, FeCl_3_, and Ca (OH)_2_, as chemical coagulants	Coagulation/Flocculation	-	99.1	84.5	96.6	-	[57]
Chitosan, as natural coagulant	Coagulation	-	80	73	92	-	[59]
Floating Hydrocotyle umbellate and Eichhornia crassipes plants	Electrocoagulation (EC)	-	-	28.2–41.9	-	Phosphorous	[62]
A polymeric-based coagulant AB121 and polyelectrolyte flocculant AB796	Coagulation/Flocculation	-	97	54	95	-	[61]

#### 3.1.4. Advanced Oxidation Processes (AOPs) for Treatment of Winery Wastewater

Advanced oxidation processes (AOPs) are increasingly applied in winery wastewater treatment due to their ability to degrade recalcitrant organic compounds and enhance biodegradability through highly reactive hydroxyl radicals (-OH). Lucas et al. [64] investigated ozone-based processes (O_3_, O_3_/UV, and O_3_/UV/H_2_O_2_) in a pilot-scale bubble column reactor, demonstrating that O_3_/UV and O_3_/UV/H_2_O_2_ significantly enhanced COD and TOC removal compared to ozonation or UV alone, largely due to the generation of reactive radicals. The study also revealed a strong dependence on initial wastewater pH, suggesting that process efficiency is closely tied to operational conditions. Similarly, Ippolito et al. [65] evaluated the Fenton reaction followed by lime neutralization and bentonite precipitation, reporting up to 54% COD reduction.

**Figure 2:**
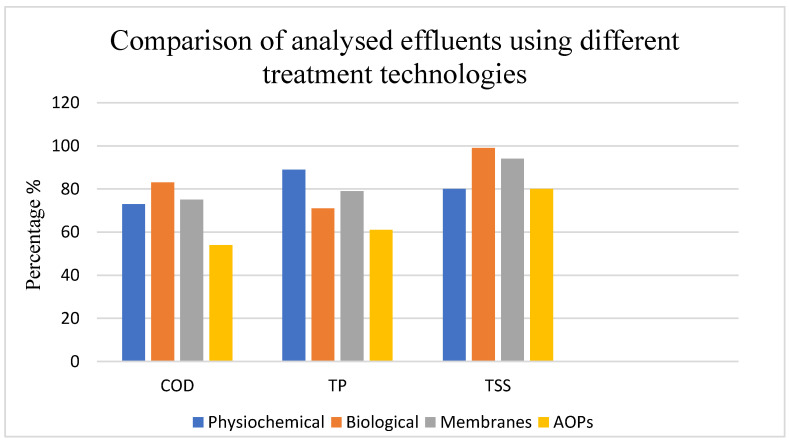
Comparison of analysed effluents using different treatment technologies. (**Physiochemical**: COD = 73% [59], TP = 82% [66], TSS = 80% [59]; **Biological**: COD = 96% [50], TP = 71% [67], TSS = 59% [67]; **Membranes**: COD = 75% [56], TP = 76% [68], TSS = 94% [68]; **AOPs**: COD = 54% [65], TP = 62% [69], TSS = 80% [66]).

When compared with other technologies (Figure 2), AOPs generally achieved lower COD removal (54%) than biological methods (>90%) but performed better than some physicochemical treatments for certain pollutants. For example, AOPs achieved 62% total phosphorus and 80% TSS removal, which positions them as complementary rather than stand-alone methods. These results suggest that AOPs are particularly valuable for targeting specific recalcitrant organics, colour, and phenolic compounds that limit the performance of conventional biological or physicochemical treatments.

The literature therefore highlights AOPs as effective polishing or hybrid treatment options rather than primary standalone methods for winery wastewater. Their efficiency is strongly influenced by wastewater composition, pH, and the presence of radical scavengers, underscoring the need for careful integration with pre-treatment steps. Future research should focus on optimizing energy consumption, lowering operational costs, and scaling up under real winery conditions to enable environmentally friendly industrial applications.

### 3.2. Limitations of Current Technologies

Although current technologies such as biological, physicochemical, membrane, and advanced oxidation processes (AOPs) offer several advantages, they also have certain limitations. For instance, aerobic biological processes require high energy input and generate a significant amount of biomass. In contrast, anaerobic biological processes are sensitive to shock loading and generate wastewater that requires additional treatment before the final discharge. Biological processes generally require a significant amount of energy (for aeration) and a substantial amount of sludge [20,70]. For physical treatment, the removal of organics from the wastewater may be challenging. The chemical treatments face difficulties, but they can quickly oxidize and breakdown the organic contaminants, making them an effective wastewater treatment approach. While membrane filtration and separation techniques are effective for treatment of winery wastewater, they also face limitations such as membrane fouling, susceptibility to chemicals, and potential for damage. Fouling, caused by the deposition of solids onto the membrane, is a major drawback, increasing operating costs and requiring frequent maintenance [71,72]. High cost is one of the shortcomings in AOPs that prevent the wide application of this treatment process in developing countries, which lies in its high operational cost due to its high energy consumption and chemical reagents. Poor solubility of ozone in water, the high energy requirement, and the generation of reaction products that could be even more hazardous than the parent compounds also limit the use of AOPs in treating WWW [65,73].

Despite the promising lab-scale results of the current treatment technologies, there is still a lack of data in the utilization of sustainable materials and green technologies in the treatment of winery wastewater. There are numerous opportunities for using innovative materials and green technology in wastewater treatment, particularly as the global focus moves to sustainability, circular economy, and climate resilience. These advances may include the use of bio-based sustainable materials to improve treatment efficiency, reduce environmental impact, and allow for resource recovery.

### 3.3. Framework for Winery Wastewater Treatment and Reuse

**Figure 3:**
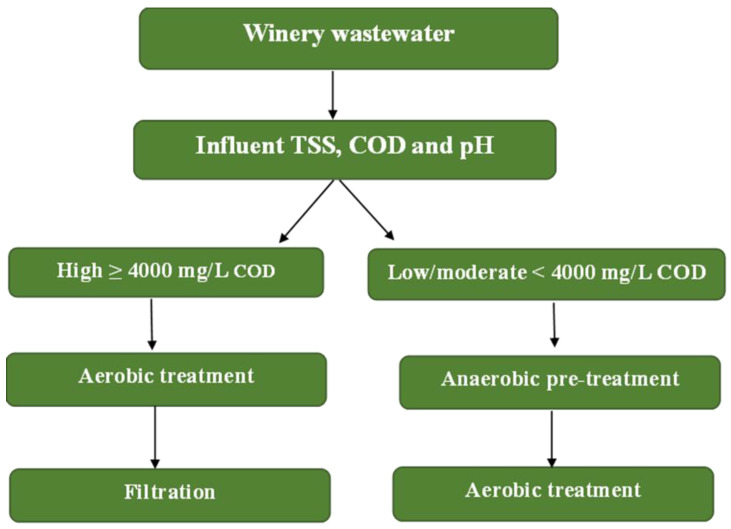
A decision-tree framework to guide the selection of treatment trains based on the influent COD, TSS, and pH.

Winery wastewater is characterized by high organic loads (in a range from 200 to 20,000 g/L), suspended solids, and acidic pH, thus making its management a significant environmental challenge that requires tailored treatment strategies [5,21,25]. As shown in Figure 3, a decision-tree framework has been proposed to guide the selection of treatment trains based on the influent COD, TSS, and pH, as well as the intended reuse target (discharge vs. irrigation). Research has shown that low strength wastewater (COD < 4000 mg/L) can be effectively treated through aerobic process with setting and filtration for reuse in irrigation [25,74], whereas high strength wastewater (COD > 4000 mg/L) generally requires anaerobic pre-treatment followed by aerobic polishing to meet discharge requirements [2,75]. Furthermore, pH correction is often necessary to maintain microbial activity, and overall treatment selection should balance pollution removal, compliance with regulatory standards, and sustainability objectives such as winery fertigation and energy recovery [5].

## 4. Bio-Based Composites for Wastewater Treatment

Bio-based composites, derived from renewable sources like plants and microorganisms, offer a sustainable approach to wastewater treatment. These may include composites with cellulose and chitosan. Chitosan and cellulose have drawn the most research interest as biopolymers for use in water purification processes, such as membrane filtration, micropollutant removal, dye degradation, and oil–water separation, due to their biocompatibility, disinfection ability, non-toxicity, and adsorption behaviour [12].

### 4.1. Properties of Cellulose and Chitosan

**Figure 4:**
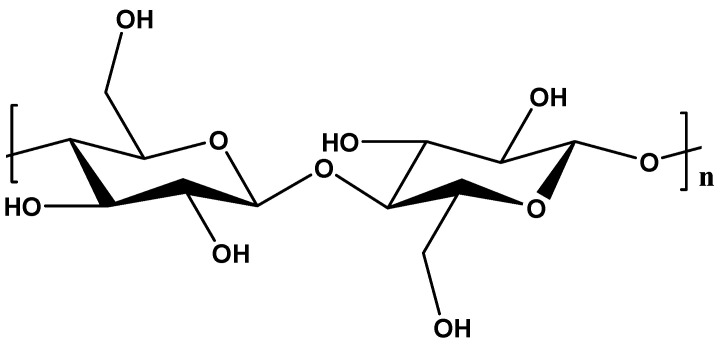
Chemical structure of cellulose.

Cellulose is known as the most abundant organic biopolymer with distinctive properties. It is made up of a long linear chain of β-1-4 d-glucose units joined together (Figure 4). Cellulose can be obtained from different sources such as wood cells, plants, and algae as well as bacteria. The linearity of the chains in cellulose is facilitated by numerous hydrogen bonds between hydroxyl groups within neighbouring chains. These hydrogen bonds create a rigid and stable crystal structure, significantly contributing to cellulose’s high tensile strength and hydrolysis resistance [76,77].

Cellulose has been used in the development of membranes and filters for water treatment applications due to its high surface area and low cost. Cellulose-based nanomaterials have been found to effectively remove contaminants such as dyes, heavy metals, and organic pollutants from water and wastewater [78]. It is known to actively interact well with water, due to the presence of the numerous hydroxyl groups along its molecular chains. However, cellulose tend to swell in water due to the presence of water molecules “packing” into the disordered regions of its semicrystalline structure [76,79,80]. To address this issue cellulose is usually incorporated with other natural biopolymers such as chitosan to enhance its properties for water treatment.

**Figure 5:**
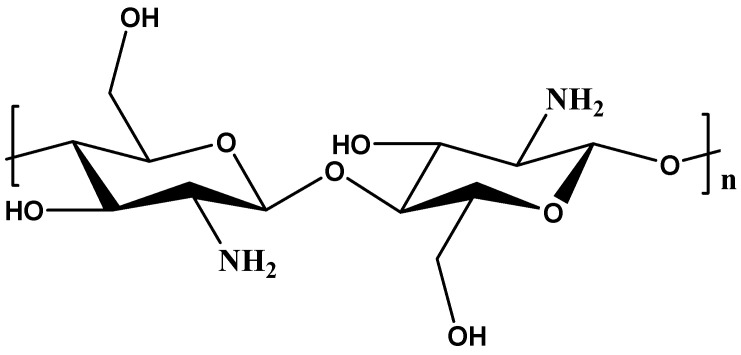
Chemical structure of chitosan.

Chitosan is a biopolymer derived from chitin, a promising and sustainable material for water treatment due to its unique properties. It has a linear structure that consists of β-(1-4)-linked D-glucosamine and N-acetyl-D-glucosamine (Figure 5). Chitosan can be found in different sources such as the exoskeletons of crustaceans (such as prawns and shrimps) and the cell walls of different fungi and insects [81,82]. Its properties such as naturally occurring, non-toxic, low-cost, biodegradable, high pollutant adsorption capacity, water adsorption capacity, and positively charged make it effective in removing various contaminants from water through adsorption and coagulation mechanisms [83]. Chitosan contains a more significant number of primary amines (–NH_2_) as well as hydroxyl (–OH) groups that provide active sites for efficient adsorption. The unique structure of chitosan offers easy removal of anionic or cationic pollutants like reactive dyes, direct dyes, acids, and metals. Due to their abundance, low cost, hydroxyl and amino groups, and capacity to extract different types of pollutants from wastewater, chitosan and its derivatives have become promising adsorbents [60]. However, its low mechanical strength, lack of selectivity, and solubility in acidic media limits its actual application [81,84].

### 4.2. Cellulose/Chitosan Composites for Treatment of Winery Wastewater

Cellulose/chitosan composites present a viable and sustainable approach to treating wastewater from wineries due to their superior adsorption capabilities, stability, biodegradability, low cost, and reusability. These composites, which are derived from agricultural waste, can be used to form various moulds like hydrogel beads, membranes, and aerogels to efficiently absorb contaminants from wastewater [85,86]. The studies summarized in Table 4 provide insights into the different mechanisms through which these materials operate, primarily adsorption, filtration, and coagulation, and their effectiveness in removing pollutants from wastewater.

Bio-based polymers such as cellulose and chitosan have been widely studied for their potential in wastewater treatment due to their abundance, biodegradability, low cost, and adsorption capacity. A study by Weng et al. [56] explored the application of ZrO_2_-reinforced bamboo cellulose membranes (BCM) for the treatment of wastewater using ultrafiltration and nanofiltration mechanisms, which combine adsorption and desorption. The results demonstrated over 80% turbidity reduction, 75% COD removal, and 88.6% removal of ammonia nitrogen. These findings emphasize the significance of cellulose-based membranes as reactive surfaces for nitrogenous and organic chemical adsorption, in addition to their physical barriers. The presence of ZrO_2_ improved the membrane’s mechanical and adsorptive properties, contributing to improvements in resistance and selectivity, as indicated by similar findings in membrane literature [87]. According to Sharma et al. [88], NOCNF achieved an 84% removal efficiency of cadmium ions (Cd^2+^) at a concentration of 250 ppm through adsorption. The high efficiency can be attributed to the large surface area and the presence of carboxylic acid groups, which provide active binding sites for positively charged metal ions. This is consistent with studies showing that surface-functionalized nanocellulose offers strong affinity for divalent metal ions [89]. These materials are especially promising for industrial applications where heavy metal contamination is a concern.

Chitosan, a natural polymer derived from chitin, has been extensively studied for its coagulation and flocculation capabilities in wastewater treatment. The study by Rizzo et al. [59] reported that chitosan achieved 80% removal of TSS, 92% turbidity reduction, and 73% COD removal in winery wastewater. These results align with chitosan’s known properties wherein its cationic amine groups interact with negatively charged particles and colloids, making it an effective natural coagulant [83]. The dual action of charge neutralization and bridging leads to efficient floc formation and pollutant capture. Importantly, chitosan offers a non-toxic and biodegradable alternative to traditional chemical coagulants, making it suitable for environmentally sensitive applications.

While each material offers unique advantages, membrane-form cellulose for filtration, functionalized nanocellulose for heavy metal adsorption, and chitosan for coagulation, their integration or combination in composite materials can yield synergistic effects. For instance, cellulose–chitosan hydrogels or films can combine the mechanical strength and high surface area of cellulose with the reactivity and flocculation capacity of chitosan. However, the literature also mentions drawbacks such as low mechanical strength, pH sensitivity, and limited selectivity, particularly for single-use or extremely variable wastewater streams. Future research should concentrate on composite design, functionalization methodologies, and pilot-scale applications to improve performance and enable real-world implementation in winery wastewater treatment.

**Table 4:** Some of the previous studies conducted using cellulose, chitosan, and their composites for treatment of wastewater.

Biopolymer/Composites	Mechanism for Pollutant Removal	COD (%)	TSS (%)	Turbidity (%)	Ions	References
ZrO_2_/BCM (bamboo cellulose membranes)	ultrafiltration and nanofiltration membrane (adsorption and desorption)	75	-	80	88.6% of (NH_4_^+^)	[56]
Nanocelluloses, in the form of carboxycellulose nanofibers (NOCNF)	Adsorption	-	-	-	84% of Cd^2+^	[88]
Chitosan	Coagulation	73	80	92	-	[59]
Chitosan	Coagulation	47	95	97	-	[90]

#### 4.2.1. Industrial Applications of Bio-Based Composite Adsorbents

The section above highlights the findings of using bio-based composites such as cellulose and chitosan at laboratory scale, these composites can further be applied in industrial wastewater treatment due to their adsorption capacity and biodegradability. This section discusses representative industrial applications and their comparative performance.

A study by Liu et al. [91] used novel EDTA-chitosan/alginate to remove lead (Pb (II)) and methyl blue in wastewater, achieving removal efficiencies of 76.6% and 80.3%, respectively. This efficiency was associated with the strong chelating ability of amino and hydroxyl functional groups on chitosan. Similarly, Elhady et al. [92] reported the use of cross-linked chitosan composite as an eco-friendly bio-adsorbent in the removal of Congo dyes (DR) in industrial wastewater. Their results revealed a remarkable efficiency of 99.7% CR removal under optimum conditions.

Cellulose based composites have also shown promising industrial outcomes. Khalid et al. [93] used magnetic chitosan/cellulose nanofiber-Fe (III) [M-Ch/CNF-Fe (III)] composites for the elimination of Cr (VI), Cu (II), and Pb (II) from aqueous solution. The results showed that Cu (II) elimination obtained was about 86%, and Cr (VI) and Pb (II) elimination values obtained were about 76% and 99%, respectively. These finding further confirm efficient removal of metals in industrial wastewater using chitosan/cellulose-based composites. At the industrial or pilot scale, these composites are gaining attention as low-cost and non-toxic alternatives to synthetic flocculants. Limited lifetime under changeable pH, and scaling costs for continuous biopolymer production are the most significant issues in these composites.

#### 4.2.2. Durability and Economic Value of Cellulose/Chitosan Composites

Cellulose/chitosan composites exhibit excellent mechanical strength and environmental durability making them suitable for applications such as wastewater treatment and sustainable irrigation systems. For instance, a study by Kusmono et al. [94] investigated the incorporation of cellulose nanocrystals (CNCs) into chitosan films. Their finding revealed that 4 wt.% of CNCs increased the tensile strength, tensile modulus, and elongation at break by 206%, 138%, and 277%, respectively. This indicated a significant enhancement in the mechanical properties of the composites [94]. Furthermore, these cellulose/chitosan composites have shown a good thermal stability, with degradation temperatures ranging between 350 and 450 °C, ensuring their reliability under various environmental conditions [95]. Cazón et al. [96] further investigated the composite films from cellulose, chitosan, and polyvinyl alcohol. Their results showed an improved toughness with values ranging from 00.47 to 8.01 MJ/m^3^, and the water adsorption values from 52.30 to 143.56%. Yadav et al. [97] explored the cellulose nanocrystal (CNC) reinforced chitosan (CS)-based UV barrier composite films. The results showed that the addition of only 4 wt.% CNC in the CS film improved the tensile strength and modulus by up to 39% and 78%, respectively. These results showed that these composites had improved mechanical properties, a high capacity to adsorb water, and good barrier properties against UV radiations, which are significantly important for water treatment applications.

The economic viability of cellulose/chitosan composites is highlighted by their low-cost production and sustainability. Cellulose, derived from renewable plant sources, and chitosan, obtained from waste such as crustacean shells, are both abundant and inexpensive materials. Their combination not only reduces material costs but also aligns with the growing demand for eco-friendly alternatives to synthetic polymers. Moreover, these composites are biodegradable, offering an environmentally responsible option for applications such as food packaging and water purification [98]. Studies showed that bio-based adsorbents cost 40–60% less to produce than activated carbon or synthetic resins, while offering comparable removal efficiency for dyes and metals [99]. The economic advantage lies not only in material cost but also in waste valorization turning agro-industrial waste (cellulose) and seafood waste (chitosan) into high-value environmental materials.

The durability and economic advantages of cellulose/chitosan composites ensure prolonged functional life in demanding applications, and their low-cost effectiveness, reusability, and eco-friendly production make them a sustainable and financially attractive option for wastewater treatment and resource recovery systems. These attributes are particularly advantageous in sectors focused on environmental remediation and sustainable agriculture.

### 4.3. Mechanism of Pollutant Removal

Wastewater treatment relies on several mechanisms for pollutant removal, including adsorption, filtration, and ion exchange. These mechanisms have been widely investigated, with varying degrees of efficiency depending on the pollutant type and operating conditions. Adsorption has received significant attention due to its ability to remove heavy metals, dyes, organic compounds, and other pollutants [100,101,102]. For instance, Jorge et al. [103] demonstrated that combining adsorption and thermos-catalytic processes causes substantial reductions in winery wastewater, with total organic carbon (76.7%), COD (81.4%), and polyphenols (over 99%) successfully removed. Such findings highlight adsorption’s versatility and high efficiency, particularly for organic pollutants. However, adsorption efficiency strongly depends on the adsorbent material, and the cost and regeneration of adsorbents remain key challenges for large-scale applications. Khan et al. [104] evaluated coal as an adsorbent for phosphate removal using adsorption as the mechanism of pollutant removal. The results of this study showed that the adsorption of phosphate ions on virgin coal was significantly higher than on charcoal, coal, and coal ash evacuated at 200 °C. This strong adsorption capability of coal is attributed to the porosity, organic carbon, and inorganic components present in the coal.

Filtration, particularly membrane-based technologies, has also been extensively studied for wastewater treatment. Techniques such as microfiltration (MF), ultrafiltration (UF), nanofiltration (NF), and reverse osmosis (RO) have been applied depending on the size of the target contaminants [105]. Qdais et al. [106] reported that RO and NF effectively removed heavy metals from synthetic wastewater, achieving 98–99% removal of Cu^2+^ and Cd^2+^ with RO and about 90% with NF. Similarly, Mohsen-Nia et al. [107] found RO to achieve 99.5% removal of Cu^2+^ and Ni^2+^. While these studies underscore the high performance of RO, its widespread application is limited by high energy demands and maintenance costs, suggesting the need for more energy-efficient alternatives.

Ion exchange has mainly been explored for the removal of hardness and heavy metals from wastewater [108,109]. For example, Tan et al. [110] investigated the use of Penicillium chrysogenium in ion exchange processes and achieved modest removal efficiencies for Cu^2+^ (4.66%), Ni^2+^ (5.45%), Zn^2+^ (11.55%), and Cr^3+^ (1.69%). Although ion exchange resins can be highly selective, their performance in real wastewater systems is often limited by competing ions and resin fouling.

In general, the literature suggests that adsorption remains the most effective for organic pollutants, while membrane-based filtration demonstrates superior performance for heavy metals. Ion exchange, though promising in concept, often shows lower efficiencies in practice. Importantly, high operational costs and energy demands continue to restrict the scalability of these technologies. Future research should therefore prioritize the development of cost-effective adsorbents, energy-efficient membranes, and hybrid systems that combine the strengths of multiple mechanisms.

## 5. Integration with Polymer Matrices

### 5.1. Overview of PAM, PVA, and PEG Properties and Applications

In the treatment of winery wastewater, polymers play a crucial role by aiding in the separation of solids and impurities, enhancing settling and filtration, and thickening sludge for easier handling and disposal. Polymers such as polyacrylamide (PAM), polyvinyl alcohol (PVA), and polyethylene glycol (PEG) have received attention in the treatment of wastewater due to their distinctive properties such as biodegradability, non-toxicity, and biocompatibility. Polyacrylamide is a synthetic water-soluble polymer with excellent properties such as high hydrophilicity, good adhesiveness, flocculation properties, and non-toxicity [14]. It can be in different physical forms such as powder, microbeads, liquid, and emulsion providing flexibility in terms of applications. It is commonly used as coagulants or flocculants in water treatment industries, due to its ability to absorb and retain water assisting with wastewater clarification and purification, facilitating the removal of pollutants easily [111].

On the other hand, PVA is a biodegradable linear polymer with hydroxyl as a functional group and carbon chains as the backbone. It is a versatile polymer with a range of desirable properties including water-solubility, high tensile strength, high flexibility, biocompatibility, good film-forming, emulsifying, and good adhesive properties [15,112]. Due to these properties, PVA can be used for a wide range of applications including paper production and water treatment. It may also be used in the fabrication of separation membranes (MF, NF, UF, and RO membranes) [113]. PEG is a synthetic polymer with various applications including pharmaceutical industries. Although PEG has not been widely used in water treatment, its solubility, biocompatibility, and capacity to alter membrane characteristics make it useful in water treatment. They are used in the production of membranes for desalination and filtration as well as in wastewater treatment procedures. The hydrophilic property of PEG increases membrane permeability, thus easing pollutant removal [16,114].

### 5.2. Comparative Summary of Each Matrix

PAM, PVA, and PEG offer roles such as formulation, PV/PEG for hydrogel, membrane platforms, and PEG for hydrophilicity and fouling mitigation (see Table 5 [115]).

Integration of polymers such as PAM, PVA, and PEG in wastewater treatment not only offers a solution to environmental concerns but also presents an alternative solution in the treatment of wastewater using less expensive and easily accessible sustainable materials. Table 6 summarizes representative studies that evaluate the performance of these polymers and their composites in removing pollutants through flocculation and adsorption mechanisms.

PAM is one of the most widely used synthetic polymers in wastewater treatment, primarily as a flocculant. In a study by Wong et al. [116], PAM showed remarkable removal efficiencies, achieving 95% turbidity reduction, 98% TSS removal, 93% COD reduction. These findings confirm PAM’s strong flocculating capability due to its high molecular weight and presence of amide groups, which enable bridging between suspended particles, resulting in dense, fast-settling flocs. In another study by Íñiguez-covarrubias et al. [117], PAM was applied to tequila vinasses, a similarly high-strength agro-industrial effluent. The results revealed a 90% reduction in settleable solids (SSs), 16.8% TDS reduction, COD removal ranging between 9.0 and 23.9%, and approximately 49.5% total solids removal. While the performance on COD and TDS was lower than in previous findings, this may reflect the complex composition and salinity of vinasse, which can limit polymer–pollutant interactions. These variations highlight the need to tailor PAM formulations and dosages to specific wastewater characteristics.

The use of PEG-modified PVA/SA hydrogel blends for wastewater treatment has also shown promising results. According to Chen et al. [118], this composite achieved a COD removal efficiency of 93.35%, which is attributed to the hydrophilicity of PEG, the film-forming capacity of PVA, and the gel stability provided by sodium alginate. The hydrogel matrix facilitates pollutant diffusion and retention through adsorption and entrapment mechanisms, making it suitable for organic pollutant removal in wastewater. Such composites are particularly relevant for low-energy, passive treatment systems, including irrigation water reuse and decentralized treatment setups. An advanced application of PEG was explored in [119], where carbon nanotubes grafted with PEG were used to adsorb phenols and heavy metals. The results demonstrated approximately 99% removal of phenol, along with simultaneous removal of metals such as Cu, Hg, Cr, Fe, Co, Ni, Al, and Pb. This exceptional performance stems from the synergistic effects of CNTs’ high surface area and PEG’s hydrophilic chains, which enhance pollutant accessibility and adsorption. The ability to target multiple classes of contaminants in a single step highlights the potential of PEG-functionalized nanomaterials in complex effluent scenarios like winery wastewater, which may contain both organic micropollutants and trace metals.

The findings in this research highlight the possibilities of synthetic polymer-based materials individually or in composites to fulfil different wastewater treatment demands. PAM excels in bulk solid–liquid separation via flocculation, making it effective for primary treatment. PVA/PEG-based hydrogels offer versatile platforms for adsorption of organics and can be engineered for controlled release or regenerative use. Meanwhile, PEG-modified nanomaterials present a next-generation solution for selective adsorption in multi-contaminant wastewater. However, as the literature points out, operational parameters like pH, pollutant concentration, and polymer dose have a major impact on removal efficiency. Furthermore, while lab-scale results are encouraging, scaling up polymer applications presents hurdles such as material regeneration, environmental toxicity (especially for non-degradable PAM), and cost. There is increasing interest in hybrid techniques that blend bio-based and synthetic polymers to improve sustainability, performance, and biodegradability.

To ease comparison of the various treatment methods, all the cleaning efficiencies reported were consolidated into Table 7 (as seen below). This table summary highlights that higher COD removal was achieved within the biological and composite-based treatment methods compared to the physicochemical treatment and advanced oxidation-based methods.

## 6. Synthesis and Characterization of Composites

### Synthesis Approaches

Various techniques, such as solution blending, melt blending, in situ polymerization, and electrospinning, are used to fabricate polymer composites for water treatment applications such as adsorption, membrane separation, and advanced oxidation processes [120,121,122,123]. The development of polymer-based composites for wastewater treatment is increasingly drawing attention due to their functional surface chemistries and high pollutant-removal efficiency. Table 8 presents a variety of studies that investigated different polymer composite systems, with an emphasis on their synthesis via solution mixing, followed by physicochemical characterization using techniques like Fourier-transform infrared spectroscopy (FTIR), scanning electron microscopy (SEM), modulated differential scanning calorimetry (MDSC), and thermogravimetric analysis (TGA).

A study by Sun et al. [124] investigated the combination of aluminium ferric sulfate (PAFS) and cationic polyacrylamide (CPAM) via solution mixing, aiming to enhance coagulation performance. FTIR analysis showed no formation of new chemical bonds, indicating a physical interaction rather than a chemical modification between the two components. SEM micrographs revealed fluffy and wrinkled surfaces on PAFS particles, which promote effective bridging and entrapment of colloidal particles during flocculation. The composite achieved 96.7% turbidity removal and 86.6% COD reduction, demonstrating high efficacy. Moreover, the sludge volume was significantly reduced, which is advantageous for post-treatment sludge handling and cost savings [125]. This result confirms the synergistic effects of inorganic–organic flocculant systems, where PAFS offers strong charge neutralization and CPAM provides bridging capabilities.

In another study by Rajeswari et al. [126], chitosan was blended with PEG and PVA through solution mixing to form composite adsorbents for nitrate removal at acidic pH of 3. SEM images revealed well-developed open pores, facilitating mass transfer and enhanced surface accessibility for nitrate ions. FTIR spectra showed shifts in amide and hydroxyl group bands, confirming hydrogen bonding between the amide carbonyl groups of chitosan and PEG’s hydroxyl groups. These findings suggest that PEG integration does not merely increase hydrophilicity but also enhances structural integrity and pollutant binding sites. The open-pore morphology and strong intermolecular interactions support high adsorption performance, especially under controlled pH conditions. In a closely related study by Perez-Calderon et al. [127], chitosan/PVA composites were further analysed for their dye adsorption potential. FTIR analysis (specifically ATR-FTIR) confirmed electrostatic interactions and hydrogen bonding between chitosan and PVA. These interactions stabilized the composite network, facilitating effective pollutant binding. The composite achieved approximately 91% dye removal at pH 2.5, showcasing its suitability for treating textile or winery effluents with colorants. Additional thermal characterization using MDSC and TGA demonstrated that the composites exhibited good thermal stability, a crucial requirement for long-term application and possible regeneration cycles.

All the polymer composites in these studies were prepared using solution mixing, a widely adopted technique due to its simplicity, low cost, and compatibility with a wide range of polymers and fillers. The use of FTIR and SEM is essential for confirming molecular interactions and surface morphology, while MDSC and TGA provide insights into thermal behaviour and composite integrity under operational stress. From the reviewed studies, it is evident that polymer composites synthesized via solution mixing exhibit strong pollutant-removal potential, particularly for turbidity, nitrate, COD, and dyes. The choice of polymers such as chitosan, PEG, PVA and their functional group interactions significantly influence performance. The incorporation of inorganic coagulants like PAFS or synthetic polymers like CPAM further enhances flocculation and reduces sludge volume, making these composites highly applicable in winery wastewater treatment scenarios. However, the effectiveness of these materials remains pH-dependent, and future studies should focus on broadening the pH range, improving mechanical durability, and scaling up production for industrial use.

**Table 8:** The previous studies on polymer composites using different preparation methods and characterization techniques.

Polymer Composites	Preparation Method	Main Findings/Observations	References
Aluminium ferric sulfate (PAFS) with cationic polyacrylamide (CPAM)	Solution mixing	FTIR results indicated no new chemical binding between PAFS and CPAM. SEM images showed that PAFS had fluffy and wrinkled surface structures, which were favourable for coagulation. The maximal flocculation efficiency: (96.7% turbidity removal rate and 86.6% COD removal rate). PAFS-CPAM flocculants further reduced the sludge volume.	[124]
Chitosan/PEG and Chitosan/PVA polymer	Solution mixing	Both composite materials successfully adsorb nitrate from aqueous solutions at pH 3. SEM micrographs showed that the surface exhibited well-developed open pores, which is due to mass transfer of nitrate ions onto the adsorbents. In (Chitosan/PEG) composites, FTIR revealed a change in chitosan amide peaks, and this was attributed to hydrogen bonding between the amide carbonyl and PEG hydroxyl.	[126]
Chitosan/PVA	Solution mixing	ATR-FTIR also confirmed the electrostatic interactions by hydrogen bonds between PVA and chitosan. Removal of dyes of about 91% was achieved at pH = 2.5. Modulated differential scanning calorimetry (MDSC) and thermogravimetric analysis (TGA) confirmed the stability of the composites.	[127]

## 7. Application for Irrigation Purposes

### 7.1. Potential Benefits and Risks of Using Treated Winery Wastewater

Benefits of using treated water may include water conservation (reuses wastewater, reducing pressure on freshwater sources), improved soil moisture, which may have been disturbed due to regular irrigation. Retaining nutrients like potassium (K), nitrogen (N), and phosphorus (P) to reduce the need for the use of synthetic fertilizers [23]. Wastewater may contain excess organic matter, which may cause oxygen depletion in soil, odours, or promote microbial imbalances. High electrical conductivity (EC) may lead to soil salinization, harming crops and reducing soil productivity (carbon soil pH). Furthermore, WWW may contain pathogens that can introduce harmful microbes. High sodium levels degrade soil structure, reducing infiltration and aeration. Crops may be affected by the residues of chemicals found in cleaning agents and additives. Lastly, the alkalinity or acidity can cause nutrient deficiency and affect soil microbial life [5,8].

#### Risk Mitigation

The following risk mitigations may arise from the treated WW for irrigation purposes. This includes the following:

- Blend treated WWW with freshwater to meet EC/SAR
- Crop selection by crop tolerance
- Seasonal rotation of reuse plots
- Quarterly metals panel
- Soil EC/SAR transect post-irrigation

### 7.2. Guidelines for Irrigation Water Quality

To operationalize these limits, treatment trains in section three should be tuned to hit pH 6.5–8.8, EC < 0.7–0.75 dS/m, SAR < 3–6, and non-detect heavy metals at the point of use. The reuse of treated winery wastewater for irrigation is governed by stringent water quality standards to safeguard soil health, crop productivity, and public safety. Table 9 presents comparative irrigation water quality guidelines from three countries—the United States, South Korea, and India—highlighting acceptable ranges for critical parameters such as pH, electrical conductivity (EC), sodium adsorption ratio (SAR), major ions, and heavy metals.

In the US, agencies such as the Environmental Protection Agency (EPA) and the United States Department of Agriculture (USDA) recommend that irrigation water maintain a pH between 6.5 and 8.4, aligning with optimal nutrient availability and microbial stability in soils. The EC threshold (<0.7 dS/m) indicates low salinity risk, essential for preventing salt buildup in arable lands. SAR values below three are considered low-risk, preserving soil permeability and structure. Sodium and chloride levels are capped at 70 mg/L and 140 mg/L, respectively, due to their potential to interfere with plant metabolism and irrigation equipment. The heavy metal limits (e.g., Pb, Cd < 0.01 mg/L; As < 0.10 mg/L) are extremely strict, reflecting concerns about crop uptake, food safety, and groundwater contamination [128].

South Korea guidelines are established by Rural Development Administration (RDA) and the Ministry of Environment follows a similar risk-averse approach, though with slightly broader pH tolerance (5.5–8.5). The EC and SAR thresholds remain conservative (<0.7 dS/m and <3, respectively), reinforcing efforts to protect soils from solidicity and salinization. Notably, South Korea specifies additional ion limits such as boron (B) < 0.5 mg/L, recognizing its phytotoxic effects on sensitive crops. Heavy metal limits mirror US standards, with particularly stringent caps on mercury (Hg < 0.001 mg/L), acknowledging its long-term ecological persistence and bioaccumulation risks [129].

Institutions such as the Central Pollution Control Board (CPCB) and the Indian Council of Agricultural Research (ICAR) provide guidelines for water quality in India. The recommended pH range (6.5–8.4) and EC limit (<0.75 dS/m) are slightly more lenient than those of South Korea, allowing for broader reuse opportunities in semi-arid zones. India allows SAR values up to 10 under the “safe” category, assuming regular soil monitoring to mitigate sodium effects. Sodium and chloride limits are comparable to those of other countries, while boron is differentiated into safe (<0.5 mg/L) and tolerable (<0.2 mg/L) thresholds, depending on crop sensitivity. The heavy metal limits are closely aligned with global best practices (e.g., Cd < 0.01 mg/L, Pb < 0.1 mg/L, Hg < 0.001 mg/L) [130,131].

These international guidelines underscore a shared recognition of the multi-dimensional risks associated with using reclaimed wastewater for irrigation including soil degradation, salinity buildup, crop toxicity, and human exposure to harmful contaminants. While the baseline standards for pH, EC, and SAR are relatively consistent, there is variation in the acceptable levels of specific ions and trace metals, reflecting differences in national crop types, soil profiles, and environmental policies. For South Africa where water scarcity and wine production are both prominent, adapting such guidelines is essential when considering treated winery wastewater for sustainable irrigation reuse.

**Table 9:** Summary of guidelines for irrigation water quality in other countries.

Country	pH	EC (dS/m)	SAR	Ions (mg/L)	Heavy Metals (mg/L)	References
United State (US)	6.5–8.4	<0.7	<3—Low risk	Chloride (Cl^−^): <140 Sodium (Na^+^): <70	Lead (Pb): <0.01 Cadmium (Cd): <0.01 Arsenic (As): <0.10	[128]
South Korea	5.5–8.5	<0.7	<3—Safe	Sodium (Na^+^): <70 Chloride (Cl^−^): <100 Boron (B): <0.5	Cadmium (Cd): <0.01 Lead (Pb): <0.05 Chromium (Cr): <0.10 Arsenic (As): <0.10 Mercury (Hg): <0.001	[129]
India	6.5–8.4	<0.75	<10 Safe	Sodium (Na^+^): <60 Chloride (Cl^−^): <140 Boron (B): <0.5 (safe), <0.2 (tolerable)	Cadmium (Cd): <0.01 Lead (Pb): <0.1 Chromium (Cr): <0.1 Arsenic (As): <0.1 Mercury (Hg): <0.001	[130,131]

In South Africa, irrigation water quality guidelines are provided by the Department of Water and Sanitation (DWS) to ensure that treated wastewater used in agriculture supports long-term soil health, crop productivity, and environmental protection [132]. Table 10 outlines these critical thresholds for physicochemical parameters, which serve as a regulatory benchmark when assessing the reuse potential of winery wastewater.

The recommended pH range is 6.5–8.4, which ensures optimal nutrient solubility and microbial activity in soils. The ideal EC value is less than 0.75 dS/m, which reflects the acceptable salinity level in irrigation water; elevated EC values can lead to salt accumulation in soils. High SAR values are associated with reduced infiltration, surface crusting, and soil compaction, which compromise water movement and root penetration, thus the ideally acceptable SAR is less than six. TDS values should remain below 450 mg/L, especially for salt-sensitive crops. Excessive South African standards impose extremely low limits on heavy metals, given their toxicity, persistence, and potential for bioaccumulation. Treated wastewater used in agriculture must be regularly tested for heavy metals, especially if the winery uses metal-based pesticides or has legacy contamination from vineyard operations. Even trace levels of cadmium (Cd), lead (Pb), or arsenic (As) can render crops unfit for human consumption and degrade soil quality irreversibly. TDS can impair seed germination, reduce plant growth, and cause ionic imbalances [23,132].

These national thresholds represent a comprehensive, precautionary framework that balances the economic value of water reuse with environmental protection goals. For treated winery wastewater, routine monitoring and treatment optimization are essential to ensure compliance. Technologies that integrate bio-based polymers and composite membranes must therefore be evaluated not only for pollutant removal efficiency but also for their ability to meet context-specific agricultural reuse standards.

## 8. Conclusions, Research Gap and Future Perspectives

The growing demand for sustainable and cost-effective winery wastewater (WWW) treatment has driven advances in bio-based polymer composites, particularly those derived from cellulose and chitosan. This is due to their biodegradability, abundance, and functional versatility, as promising natural matrices. When combined with synthetic polymers such as PAM, PVA, and PEG, these systems exhibit enhanced structural integrity, chemical stability, and targeted pollutant removal. Evidence across studies demonstrates that cellulose/chitosan-based composites consistently achieve >80% removal efficiencies for chemical oxygen demand (COD), turbidity, ammonia, and heavy metals, with some formulations achieving COD and TSS reductions above 90%. Their performance is further strengthened by hybridization with synthetic polymers through methods such as solution mixing and in situ polymerization.

### 8.1. Research Gap and Limitations

Despite notable progress, several gaps remain. Most existing studies are limited to laboratory- or bench-scale experiments under controlled conditions, with less validation in real winery environments where seasonal variability, fluctuating organic loads, and pH instability affect treatment performance. There is a lack of standardized protocols for evaluating performance, regeneration, and leachate composition of bio-based composites. Polymer leaching and long-term stability of PAM/PVA/PEG-modified materials remain underexplored, especially regarding environmental safety. Economic feasibility (ZAR/m^3^ treatment cost) and life-cycle analyses (LCAs) are limited, constraining realistic evaluation of cost-effectiveness and scalability. Few studies have examined the long-term impacts of irrigation reuse, including effects on soil EC, SAR, and crop productivity.

### 8.2. Future Perspectives

Future research should prioritize optimizing composite formulations for multi-contaminant removal, advancing green synthesis approaches, and validating performance under real-field conditions. Moreover, integrating bio-based composites into decentralized and modular treatment systems could close the water loop in the wine industry, enhance agricultural resilience in water-scarce regions, and support compliance with evolving environmental regulations. While current evidence remains largely limited to laboratory-scale investigations, scaling up and conducting economic assessments will be critical steps toward practical implementation.

Future studies should include the following:

- Pilot L/s scale hybrid trains under vintage peak loads with ZAR/m^3^ costing.
- Standardized regeneration and leachate for PAM/PVA/PEG-bio-composites.
- Soil health trials quantifying EC/SAR drift and crop response over >2 seasons.

In summary, bio-based cellulose/chitosan composites blended with PAM, PVA, and PEG represent a sustainable and innovative pathway for winery wastewater treatment. With continued research and supportive policy frameworks, these emerging materials hold the potential to transition from promising technologies to standard practice in the wine industry’s environmental management.

## Figures and Tables

**Table 1:** Typical winery wastewater ranges reported in the literature and parameters most critical for irrigation reuse.

Parameters	Typical Range	Irrigation Use (Target/Limit)	References
pH	3–4	6.5–8	[21,22]
COD	1000–20,000 mg/L	75–400 mg/L	[21,23,24]
BOD	1000–6000 mg/L	30–40 mg/L	[25]
EC	0.5–3.5 dS/m	0.75–2.0 dS/m	[22]
SAR	2–20	Less than 2	[26]
Metals (e.g Zn, Al, Fe, Pb, CD, etc.)	0.001–2 mg/L	5 mg/L	[23,26,27,28]

**Table 5:** Matrix selection for winery wastewater.

Matrix	Sweet Spot	Watch-Outs
PAM	TSS/turbidity, primary clarification	Residual PAM management; charge pH sensitivity
PVA/PEG hydrogels	Organics/some ions via adsorption–entrapment	Mechanical stability; regeneration chemistry
PEG-modified membranes	Flux, antifouling	PEG leaching; long-term stability

**Table 6:** Presents some previous work conducted using polymers to treat wastewater.

Polymer or Polymer/Composites	Method Used	Parameters Observed	Main Findings	References
PAM	Flocculation	Turbidity, TSS, sludge volume and COD	95% of turbidity reduction was achieved, 98% of TSS removal, about 93% of COD reduction, and sludge volume index (SVI) of 14 mL g^−1^ at the optimum dosage of 5 mg L^−1^.	[116]
PAM in tequila vinasses	Flocculation	Settleable solids (SSs), TSS, total dissolved solids (TDS) and COD	The SS removal efficiency was about 90%, the TDS removal efficiency was 16.8%, and the COD removal efficiency was between 9.0 and 23.9%. Removal of about 49.5% of total solids	[117]
PEG-modified-Polyvinyl alcohol (PVA)/Sodium alginate (SA) hydrogel	Adsorption	COD	COD removal rate of 93.35%.	[118]
Carbon nanotube (CNT) and grafted with polyethylene glycol (PEG)	Adsorption	Phenols	Approximately 99% of phenol removal simultaneously with Cu, Hg, Cr, Fe, Co, Ni, Al, and Pb was achieved.	[119]

**Table 7:** Cleaning efficiencies reported for the various winery wastewater treatment approaches and composite materials.

Treatment Methods	Mechanism	Processes	COD (% Removal)	Turbidity (% Removal)	TSS (% Removal)	Other Parameters Removal	References
Air-microbubble bioreactor (AMBB)	Biological	Aerobic	99	_	_	Efficient oxygen transfer	[45]
Jet-loop activated sludge reactor (JLR)	Biological	Aerobic	>90	_	_	High turbulence and low energy demand	[47]
Expanded granular sludge bed (EGSB) reactor	Biological	Anaerobic	96	_	_	Biogas generation	[50]
Chitosan coagulant	Physicochemical (coagulation)	coagulation	73	92	80	Natural polymer coagulant	[59]
FeSO_4_, Al_2_(SO_4_)_3_, FeCl_3_ coagulants	Physicochemical	chemical flocculation	84.5	96.6	99.1	_	[57]
ZrO_2_/Bamboo cellulose membrane	Bio-composite	filtration and adsorption	75	80	_	NH_4_^+^ removal = 88.6%	[56]
Chitosan/Cellulose hydrogel	Bio-composite	Adsorption and flocculation	73–95	92–97	80–97	_	[60]
PEG–PVA/SA hydrogel	Polymer composite	Adsorption	93.35	_	_	Organic matter removal	[118]
CNT–PEG composite	Nanocomposite	Adsorption	_	_	_	Removal of phenol is ≈99%; metals (such as Cu, Hg, Cr, Pb, etc.) are removed simultaneously	[119]

**Table 10:** Summarizes the guidelines for irrigation water quality in South Africa.

Parameter	Guideline Value	Important Description
Electrical Conductivity (EC)	<0.75 dS/m (ideal)	Indicates salinity; high EC can lead to salt accumulation in the soil
Sodium Adsorption Ratio (SAR)	<6 (ideal)	High SAR can degrade soil structure and reduce infiltration
pH	6.5–8.4	Affects nutrient availability and the solubility of toxic elements
Total Dissolved Solids (TDSs)	<450 mg/L (ideal)	High TDS can harm salt-sensitive crops
Chloride (Cl^−^)	<140 mg/L	High chloride levels can damage crops, especially in drip irrigation
Nitrate (NO_3_^−^)	<10 mg/L	It can be a nutrient, but excess may harm plants or lead to groundwater pollution
Heavy Metals (e.g., Cd, Pb, As)	Extremely low limits	Toxic to plants and unsafe for food crops; requires regular monitoring

## Data Availability

No new data were created or analyzed in this study. Data sharing is not applicable to this article.

## Abbreviations

The following abbreviations are used in this manuscript:

PAM	Polyacrylamide
PVA	Polyvinyl Alcohol
PEG	Polyethylene glycol
WWW	Winery wastewater
COD	Chemical oxygen demand
BOD	Biological oxygen demand
TSSs	Total suspended solids
TDSs	Total dissolved solids
AOPs	Advanced oxidation processes
EC	Electrocoagulation
Pd	Lead
SAR	Sodium adsorption ratio
RO	Reverse osmosis
MF	Microfiltration
UF	Ultrafiltration
NF	Nanofiltration

## References

[1] Graham P.M. (2025). The State of Wastewater Management in South Africa: Data Gaps, Missing Wastewater, and Green Drop Reporting. Water.

[2] Bolzonella D., Papa M., Da Ros C., Anga Muthukumar L., Rosso D. (2019). Winery Wastewater Treatment: A Critical Overview of Advanced Biological Processes. Crit. Rev. Biotechnol..

[3] Sikhau T. (2022). Soil Chemical and Microbiological Responses to Irrigation with Diluted Winery Wastewater in a Shiraz Vineyard in Stellenbosch, Western Cape. Ph.D. Thesis.

[4] Carbon S. (2014). Soil Carbon.

[5] Mosse K.P.M., Patti A.F., Christen E.W., Cavagnaro T.R. (2011). Review: Winery Wastewater Quality and Treatment Options in Australia. Aust. J. Grape Wine Res..

[6] Iloms E., Ololade O.O., Ogola H.J.O., Selvarajan R. (2020). Investigating Industrial Effluent Impact on Municipal Wastewater Treatment Plant in Vaal, South Africa. Int. J. Environ. Res. Public Health.

[7] Jorge N., Teixeira A.R., Gomes A., Peres J.A., Lucas M.S. (2023). Winery Wastewater: Challenges and Perspectives. Eng. Proc..

[8] Saraiva A., Rodrigues G., Mamede H., Silvestre J., Dias I., Feliciano M., Oliveira E Silva P., Oliveira M. (2020). The Impact of the Winery’s Wastewater Treatment System on the Winery Water Footprint. Water Sci. Technol..

[9] Vlotman D.E., Key D., Bladergroen B.J. (2022). Technological Advances in Winery Wastewater Treatment: A Comprehensive Review. S. Afr. J. Enol. Vitic..

[10] Hirzel D.R., Steenwerth K., Parikh S.J., Oberholster A. (2017). Impact of Winery Wastewater Irrigation on Soil, Grape and Wine Composition. Agric. Water Manag..

[11] Muyen Z., Moore G.A., Wrigley R.J. (2011). Soil Salinity and Sodicity Effects of Wastewater Irrigation in South East Australia. Agric. Water Manag..

[12] Khedulkar A.P., Bobade R.G., Doong R.-a., Pandit B., Ky N.M., Ambare R.C., Hoang T.D., Kumar K.J. (2025). Bio-Based Nanomaterials as Effective, Friendly Solutions and Their Applications for Protecting Water, Soil, and Air. Mater. Today Chem..

[13] Olivera S., Muralidhara H.B., Venkatesh K., Guna V.K., Gopalakrishna K., Kumar K.Y. (2016). Potential Applications of Cellulose and Chitosan Nanoparticles/Composites in Wastewater Treatment: A Review. Carbohydr. Polym..

[14] Voronova M.I., Surov O.V., Afineevskii A.V., Zakharov A.G. (2020). Properties of Polyacrylamide Composites Reinforced by Cellulose Nanocrystals. Heliyon.

[15] Sharma P., Kumar Agrawal P., Singh V.K., Chauhan S., Bhaskar J. (2023). Jitendra Bhaskar (2023) A Comprehensive Review on Properties of Polyvinyl Alcohol (PVA) Crosslinked with Carboxylic Acid. J. Mater. Environ. Sci.

[16] Sosiati H., Hanafi L.P.I., Takiyudin K.R., Harimurti S., Yusmaniar Y. (2025). Enhancing Hydrophilicity and Efficiency of PVC-Based Nanofiber Membranes by Adding PEG, Chitosan, and Silver Nanoparticles for Water Filtration. Eng. Proc..

[17] Yang C., Han Y., Tian X., Sajid M., Mehmood S., Wang H., Li H. (2024). Phenolic Composition of Grape Pomace and Its Metabolism. Crit. Rev. Food Sci. Nutr..

[18] Maicas S. (2021). Advances in Wine Fermentation. Fermentation.

[19] Laurenson S., Houlbrooke D. (2011). Winery Wastewater Irrigation, Marlborough District Council.

[20] Miklas V., Touš M., Miklasová M., Máša V., Horňák D. (2022). Winery Wastewater Treatment Technologies: Current Trends and Future Perspective. Chem. Eng. Trans..

[21] Malandra L., Wolfaardt G., Zietsman A., Viljoen-Bloom M. (2003). Microbiology of a Biological Contactor for Winery Wastewater Treatment. Water Res..

[22] Ayers R.S., Westcot D.W. (1985). Water Quality for Agriculture.

[23] Holmes S. (1996). South African Water Quality South African Water Quality Guidelines.

[24] Howell C.L., Myburgh P.A. (2018). Management of Winery Wastewater by Re-Using It for Crop Irrigation—A Review. S. Afr. J. Enol. Vitic..

[25] Bustamante M.A., Paredes C., Moral R., Moreno-Caselles J., Pérez-Espinosa A., Pérez-Murcia M.D. (2005). Uses of Winery and Distillery Effluents in Agriculture: Characterisation of Nutrient and Hazardous Components. Water Sci. Technol..

[26] Conradie A., Sigge G.O., Cloete T.E. (2015). Influence of Winemaking Practices on the Characteristics of Winery Wastewater and Water Usage of Wineries. S. Afr. J. Enol. Vitic..

[27] Bustamante M.A., Moral R., Paredes C., Pérez-Espinosa A., Moreno-Caselles J., Pérez-Murcia M.D. (2008). Agrochemical Characterisation of the Solid By-Products and Residues from the Winery and Distillery Industry. Waste Manag..

[28] Howell C.L., Myburgh P.A., Lategan E.L., Hoffman J.E. (2016). Seasonal Variation in Composition of Winery Wastewater in the Breede River Valley with Respect to Classical Water Quality Parameters. S. Afr. J. Enol. Vitic..

[29] Vlotman D., Key D., Cerff B., Bladergroen B.J. (2024). Wastewater Treatment Using Shear Enhanced Flotation Separation Technology: A Pilot Plant Study for Winery Wastewater Processing. Processes.

[30] Ioannou L.A., Puma G.L., Fatta-Kassinos D. (2015). Treatment of Winery Wastewater by Physicochemical, Biological and Advanced Processes: A Review. J. Hazard. Mater..

[31] Mulidzi A.R., Clarke C.E., Myburgh P.A. (2019). Response of Soil Chemical Properties to Irrigation with Winery Wastewater on a Well-Drained Sandy Soil. S. Afr. J. Enol. Vitic..

[32] Mulidzi A.R. (2016). The Effect of Winery Wastewater Irrigation on the Properties of Selected Soils from the South African Wine Region. Ph.D. Thesis.

[33] Mulidzi A.R., Clarke C.E., Myburgh P.A. (2015). Effect of Irrigation with Diluted Winery Wastewater on Cations and PH in Four Differently Textured Soils. S. Afr. J. Enol. Vitic..

[34] Sánchez M., Gonzalo O.G., Yáñez S., Ruiz I., Soto M. (2021). Influence of Nutrients and PH on the Efficiency of Vertical Flow Constructed Wetlands Treating Winery Wastewater. J. Water Process Eng..

[35] Xie Y., Ning H., Zhang X., Zhou W., Xu P., Song Y., Li N., Wang X., Liu H. (2024). Reducing the Sodium Adsorption Ratio Improves the Soil Aggregates and Organic Matter in Brackish-Water-Irrigated Cotton Fields. Agronomy.

[36] Van Schoor L. (2005). Guidelines for the Management of Wastewater and Solid Waste at Existing Wineries.

[37] Simhayov R., Ohana-Levi N., Shenker M., Netzer Y. (2023). Effect of Long-Term Treated Wastewater Irrigation on Soil Sodium Levels and Table Grapevines’ Health. Agric. Water Manag..

[38] Mulidzi A.R. (2001). Environmental Impact of Winery Effluent in the Western and Northern Cape Provinces. Ph.D. Thesis.

[39] Malandra L. (2003). Biodegradation of Winery Wastewater. Ph.D. Thesis.

[40] Riera F.S., Brümmer B. (2022). Environmental Efficiency of Wine Grape Production in Mendoza, Argentina. Agric. Water Manag..

[41] Matos C., Castro M., Baptista J., Valente A., Briga-Sá A. (2024). The Use of Water in Wineries: A Review. Sci. Total Environ..

[42] Nirmal N., Mahale K.R., Rathod N.B., Siddiqui S.A., Dhar B.K. (2025). Winery Waste: A Sustainable Approach for Bioactive Compound Extraction and Various Industrial Applications. Process Saf. Environ. Prot..

[43] Andreottola G., Foladori P., Ziglio G. (2009). Biological Treatment of Winery Wastewater: An Overview. Water Sci. Technol..

[44] Alayu E., Leta S. (2021). Evaluation of Irrigation Suitability Potential of Brewery Effluent Post Treated in a Pilot Horizontal Subsurface Flow Constructed Wetland System: Implications for Sustainable Urban Agriculture. Heliyon.

[45] Oliveira M., Queda C., Duarte E. (2009). Aerobic Treatment of Winery Wastewater with the Aim of Water Reuse. Water Sci. Technol..

[46] Skouteris G., Rodriguez-Garcia G., Reinecke S.F., Hampel U. (2020). The Use of Pure Oxygen for Aeration in Aerobic Wastewater Treatment: A Review of Its Potential and Limitations. Bioresour. Technol..

[47] Petruccioli M., Cardoso Duarte J., Eusebio A., Federici F. (2002). Aerobic Treatment of Winery Wastewater Using a Jet-Loop Activated Sludge Reactor. Process Biochem..

[48] Yildiz E., Keskinler B., Pekdemir T., Akay G., Nuhoǧlu A. (2005). High Strength Wastewater Treatment in a Jet Loop Membrane Bioreactor: Kinetics and Performance Evaluation. Chem. Eng. Sci..

[49] Sravan J.S., Matsakas L., Sarkar O. (2024). Advances in Biological Wastewater Treatment Processes: Focus on Low-Carbon Energy and Resource Recovery in Biorefinery Context. Bioengineering.

[50] Petropoulos E., Cuff G., Huete E., Garcia G., Wade M., Spera D., Aloisio L., Rochard J., Torres A., Weichgrebe D. (2016). Investigating the Feasibility and the Limits of High Rate Anaerobic Winery Wastewater Treatment Using a Hybrid-EGSB Bio-Reactor. Process Saf. Environ. Prot..

[51] Cruz-Salomón A., Ríos-Valdovinos E., Pola-Albores F., Lagunas-Rivera S., Meza-Gordillo R., Ruíz-Valdiviezo V.M., Cruz-Salomón K.C. (2019). Expanded Granular Sludge Bed Bioreactor in Wastewater Treatment. Glob. J. Environ. Sci. Manag..

[52] Donoso-Bravo A., Rosenkranz F., Valdivia V., Torrijos M., Ruiz-Filippi G., Chamy R. (2009). Anaerobic Sequencing Batch Reactor as an Alternative for the Biological Treatment of Wine Distillery Effluents. Water Sci. Technol..

[53] Shao X., Peng D., Teng Z., Ju X. (2008). Treatment of Brewery Wastewater Using Anaerobic Sequencing Batch Reactor (ASBR). Bioresour. Technol..

[54] Tapia-Quirós P., Montenegro-Landívar M.F., Reig M., Vecino X., Saurina J., Granados M., Cortina J.L. (2022). Integration of Membrane Processes for the Recovery and Separation of Polyphenols from Winery and Olive Mill Wastes Using Green Solvent-Based Processing. J. Environ. Manag..

[55] Bottino A., Capannelli G., Comite A., Jezowska A., Pagliero M., Costa C., Firpo R. (2020). Treatment of Olive Mill Wastewater through Integrated Pressure-Driven Membrane Processes. Membranes.

[56] Weng R., Chen G., He X., Qin J., Dong S., Bai J., Li S., Zhao S. (2024). The Performance of Cellulose Composite Membranes and Their Application in Drinking Water Treatment. Polymers.

[57] Braz R., Pirra A., Lucas M.S., Peres J.A. (2010). Combination of Long Term Aerated Storage and Chemical Coagulation/Flocculation to Winery Wastewater Treatment. Desalination.

[58] Gregory J., Duan J. (2001). Hydrolyzing Metal Salts as Coagulants. Pure Appl. Chem..

[59] Rizzo L., Lofrano G., Belgiorno V. (2010). Olive Mill and Winery Wastewaters Pre-Treatment by Coagulation with Chitosan. Sep. Sci. Technol..

[60] Atangana E., Ajiboye T.O., Mafolasire A.A., Ghosh S., Hakeem B. (2025). Adsorption of Organic Pollutants from Wastewater Using Chitosan-Based Adsorbents. Polymers.

[61] Vlotman D., Key D., Cerff B., Bladergroen B.J. (2023). Shear Enhanced Flotation Separation Technology in Winery Wastewater Treatment. Water.

[62] Kirzhner F., Zimmels Y., Shraiber Y. (2008). Combined Treatment of Highly Contaminated Winery Wastewater. Sep. Purif. Technol..

[63] Boinpally S., Kolla A., Kainthola J., Kodali R., Vemuri J. (2023). A State-of-the-Art Review of the Electrocoagulation Technology for Wastewater Treatment. Water Cycle.

[64] Lucas M.S., Peres J.A., Li Puma G. (2010). Treatment of Winery Wastewater by Ozone-Based Advanced Oxidation Processes (O3, O3/UV and O3/UV/H2O2) in a Pilot-Scale Bubble Column Reactor and Process Economics. Sep. Purif. Technol..

[65] Ippolito N.M., Zueva S.B., Ferella F., Corradini V., Baturina E.V., Vegliò F. (2021). Treatment of Waste Water from a Winery with an Advanced Oxidation Process (AOP). IOP Conf. Ser. Earth Environ. Sci..

[66] Melchiors E., Freire F.B. (2023). Winery Wastewater Treatment: A Systematic Review of Traditional and Emerging Technologies and Their Efficiencies. Environ. Process..

[67] Tsolcha O.N., Tekerlekopoulou A.G., Akratos C.S., Aggelis G., Genitsaris S., Moustaka-Gouni M., Vayenas D.V. (2017). Biotreatment of Raisin and Winery Wastewaters and Simultaneous Biodiesel Production Using a Leptolyngbya-Based Microbial Consortium. J. Clean. Prod..

[68] Ioannou L.A., Michael C., Vakondios N., Drosou K., Xekoukoulotakis N.P., Diamadopoulos E., Fatta-Kassinos D. (2013). Winery Wastewater Purification by Reverse Osmosis and Oxidation of the Concentrate by Solar Photo-Fenton. Sep. Purif. Technol..

[69] Orescanin V., Kollar R., Nad K., Mikelic I.L., Gustek S.F. (2013). Treatment of Winery Wastewater by Electrochemical Methods and Advanced Oxidation Processes. J. Environ. Sci. Health Part A.

[70] Gopalakrishnan G., Jeyakumar R.B., Somanathan A. (2023). Challenges and Emerging Trends in Advanced Oxidation Technologies and Integration of Advanced Oxidation Processes with Biological Processes for Wastewater Treatment. Sustainability.

[71] Muro C., Riera F., del Carmen Diaz M. (2012). Membrane Separation Process in Wastewater Treatment of Food Industry. Food Industrial Processes—Methods and Equipment.

[72] Arriagada-Carrazana J.P., Sáez-Navarrete C., Bordeu E. (2005). Membrane Filtration Effects on Aromatic and Phenolic Quality of Cabernet Sauvignon Wines. J. Food Eng..

[73] Romani A., Pinelli P., Ieri F., Bernini R. (2016). Sustainability, Innovation, and Green Chemistry in the Production and Valorization of Phenolic Extracts from *Olea europaea* L.. Sustainability.

[74] Garcia Batres J.C. (2012). Renewable Energy Extraction From Organic Winery Wastes Through Anaerobic Treatment. Ph.D. Thesis.

[75] Bolzonella D., Fatone F., Pavan P., Cecchi F. (2010). Application of a Membrane Bioreactor for Winery Wastewater Treatment. Water Sci. Technol..

[76] Darmenbayeva A., Zhussipnazarova G., Rajasekharan R., Massalimova B., Zharlykapova R., Nurlybayeva A., Mukazhanova Z., Aubakirova G., Begenova B., Manapova S. (2025). Applications and Advantages of Cellulose–Chitosan Biocomposites: Sustainable Alternatives for Reducing Plastic Dependency. Polymers.

[77] Kumalo F.I., Malimabe M.A., Mosoabisane M.F.T., Gumede T.P. (2025). Development and Characterization of PBS/EA Cellulose and PCL/EA Cellulose Biocomposites: Structural, Morphological, and Thermal Insights for Sustainable Applications. Polymers.

[78] Jiang S., Xi J., Dai H., Wu W., Xiao H. (2022). Multifunctional Cellulose Paper-Based Materials and Their Application in Complex Wastewater Treatment. Int. J. Biol. Macromol..

[79] Etale A., Onyianta A.J., Turner S.R., Eichhorn S.J. (2023). Cellulose: A Review of Water Interactions, Applications in Composites, and Water Treatment. Chem. Rev..

[80] Akter M., Bhattacharjee M., Dhar A.K., Rahman F.B.A., Haque S., Ur Rashid T.U., Kabir S.M.F. (2021). Cellulose-Based Hydrogels for Wastewater Treatment: A Concise Review. Gels.

[81] Bhatt P., Joshi S., Bayram G.M.U., Khati P., Simsek H. (2023). Developments and Application of Chitosan-Based Adsorbents for Wastewater Treatments. Environ. Res..

[82] Motshabi N., Lenetha G.G., Malimabe M.A., Gumede T.P. (2025). Cellulose Nanofibril-Based Biodegradable Polymers from Maize Husk: A Review of Extraction, Properties, and Applications. Polymers.

[83] Samoila P., Humelnicu A.C., Ignat M., Cojocaru C., Harabagiu V. (2019). Chitin and Chitosan for Water Purification.

[84] Chelu M., Musuc A.M., Popa M., Calderon Moreno J.M. (2023). Chitosan Hydrogels for Water Purification Applications. Gels.

[85] Da Róz A.L., Leite F.L., Pereiro L.V., Nascente P.A.P., Zucolotto V., Oliveira O.N., Carvalho A.J.F. (2010). Adsorption of Chitosan on Spin-Coated Cellulose Films. Carbohydr. Polym..

[86] Strnad S. (2023). Cellulose—Chitosan Functional Biocomposites. Polymers.

[87] Bekchanov D., Mukhamediev M., Eshtursunov D., Lieberzeit P., Su X. (2024). Cellulose- and Starch-Based Functional Materials for Efficiently Wastewater Treatment. Polym. Adv. Technol..

[88] Sharma P.R., Chattopadhyay A., Sharma S.K., Geng L., Amiralian N., Martin D., Hsiao B.S. (2018). Nanocellulose from Spinifex as an Effective Adsorbent to Remove Cadmium(II) from Water. ACS Sustain. Chem. Eng..

[89] Singh A., Vijayan J.G., Moodley K.G. (2020). Handbook of Nanocelluloses.

[90] Jorge N., Teixeira A.R., Marchão L., Lucas M.S., Peres J.A. (2022). Plants as Natural Organic Coagulant Powders for Winery Wastewater Treatment. Biol. Life Sci. Forum.

[91] Liu X., Ren W., Song W., Zhang W., Wang Y., Wang Y., Fan G., Zhang L., Huang Y. (2025). Novel EDTA-Chitosan/Alginate Porous Composite Beads for the Removal of Pb(II) and Methylene Blue from Aqueous Solutions. RSC Adv..

[92] Elhady S., Bassyouni M., Elshikhiby M.Z., Saleh M.Y., Elzahar M.H. (2024). Optimization of Anionic Dye Removal Using Cross-Linked Chitosan Composite as Eco-Friendly Bio-Adsorbent. Appl. Water Sci..

[93] Khalid A.M., Hosain M.S., Khalil N.A., Zulkifl M., Arafath M.A., Shaharun M.Z., Ayub R., Yahaya A.N.A., Ismail N. (2023). Adsorptive Elimination of Heavy Metals from Aqueous Solution Using Magnetic Chitosa/Cellullose-Fe(III) Composites as Bio-Sorbent. Nanomaterials.

[94] (2021). Kusmono; Wildan, M.W.; Lubis, F.I. Fabrication and Characterization of Chitosan/Cellulose Nanocrystal/Glycerol Bio-Composite Films. Polymers.

[95] Varma R., Vasudevan S. (2024). Synthesis of Composite Films Using Polymer Blends of Chitosan and Cellulose Nanocrystals from Marine Origin. J. Mater. Sci. Mater. Eng..

[96] Cazón P., Vázquez M., Velazquez G. (2018). Composite Films of Regenerate Cellulose with Chitosan and Polyvinyl Alcohol: Evaluation of Water Adsorption, Mechanical and Optical Properties. Int. J. Biol. Macromol..

[97] Yadav M., Behera K., Chang Y.H., Chiu F.C. (2020). Cellulose Nanocrystal Reinforced Chitosan Based UV Barrier Composite Films for Sustainable Packaging. Polymers.

[98] Yu Z., Ji Y., Bourg V., Bilgen M., Meredith J.C. (2020). Chitin- and Cellulose-Based Sustainable Barrier Materials: A Review. Emergent Mater..

[99] Foo K.Y., Hameed B.H. (2010). Insights into the Modeling of Adsorption Isotherm Systems. Chem. Eng. J..

[100] Akhtar M.S., Ali S., Zaman W. (2024). Innovative Adsorbents for Pollutant Removal: Exploring the Latest Research and Applications. Molecules.

[101] Satyam S., Patra S. (2024). Innovations and Challenges in Adsorption-Based Wastewater Remediation: A Comprehensive Review. Heliyon.

[102] Trivedi Y., Sharma M., Mishra R.K., Sharma A., Joshi J., Gupta A.B., Achintya B., Shah K., Vuppaladadiyamd A.K. (2025). Biochar Potential for Pollutant Removal during Wastewater Treatment: A Comprehensive Review of Separation Mechanisms, Technological Integration, and Process Analysis. Desalination.

[103] Jorge N., Teixeira A.R., Guimarães V., Lucas M.S., Peres J.A. (2022). Treatment of Winery Wastewater with a Combination of Adsorption and Thermocatalytic Processes. Processes.

[104] Khan S., Ishaq M., Ahmad I., Hussain S., Ullah H. (2013). Evaluation of Coal as Adsorbent for Phosphate Removal. Arab. J. Geosci..

[105] Ezugbe E.O., Rathilal S. (2020). Membrane Technologies in Wastewater Treatment: A Review. Membranes.

[106] Qdais H.A., Moussa H. (2004). Removal of Heavy Metals from Wastewater by Membrane Processes: A Comparative Study. Desalination.

[107] Mohsen-Nia M., Montazeri P., Modarress H. (2007). Removal of Cu^2+^ and Ni^2+^ from Wastewater with a Chelating Agent and Reverse Osmosis Processes. Desalination.

[108] Inglezakis V.J. (2010). Ion Exchange and Adsorption Fixed Bed Operations for Wastewater Treatment-Part I: Modeling Fundamentals and Hydraulics Analysis. J. Eng. Stud. Res..

[109] Ahmad H.W., Bibi H.A., Chandrasekaran M., Ahmad S., Kyriakopoulos G.L. (2024). Sustainable Wastewater Treatment Strategies in Effective Abatement of Emerging Pollutants. Water.

[110] Tan T., Cheng P. (2003). Biosorption of Metal Ions with Penicillium Chrysogenum. Appl. Biochem. Biotechnol. Part A Enzym. Eng. Biotechnol..

[111] Cheng Y.C., Wang C.P., Liu K.Y., Pan S.Y. (2024). Towards Sustainable Management of Polyacrylamide in Soil-Water Environment: Occurrence, Degradation, and Risk. Sci. Total Environ..

[112] Gaaz T.S., Sulong A.B., Akhtar M.N., Kadhum A.A.H., Mohamad A.B., Al-Amiery A.A., McPhee D.J. (2015). Properties and Applications of Polyvinyl Alcohol, Halloysite Nanotubes and Their Nanocomposites. Molecules.

[113] Vatanpour V., Teber O.O., Mehrabi M., Koyuncu I. (2023). Polyvinyl Alcohol-Based Separation Membranes: A Comprehensive Review on Fabrication Techniques, Applications and Future Prospective. Mater. Today Chem..

[114] Checchetto R. (2025). Polyethylene Glycol (PEG) Additive in Polymer Membranes for Carbon Dioxide Separation: A Critical Review on Performances and Correlation with Membrane Structure. Separations.

[115] Montalvo S., Guerrero L., Borja R., Sánchez E., Milán Z., Cortés I., Angeles de la la Rubia M. (2012). Application of Natural Zeolites in Anaerobic Digestion Processes: A Review. Appl. Clay Sci..

[116] Wong S.S., Teng T.T., Ahmad A.L., Zuhairi A., Najafpour G. (2006). Treatment of Pulp and Paper Mill Wastewater by Polyacrylamide (PAM) in Polymer Induced Flocculation. J. Hazard. Mater..

[117] Íñiguez-covarrubias G., Peraza-luna F., De Guadalajara U., De D., Agujas L., Apartado J., Guadalajara P., Miguel G., Tlajomulco C. (2007). Reduction of Solids and Organic Load Concentrations in Tequila Vinasses Using a Polyacrylamide (PAM) Polymer Flocculant.

[118] Chen Q., Ding Q., Li W., Deng J., Lin Q., Li J. (2022). Enhanced Treatment of Organic Matters in Starch Wastewater through Bacillus Subtilis Strain with Polyethylene Glycol-Modified Polyvinyl Alcohol/Sodium Alginate Hydrogel Microspheres. Bioresour. Technol..

[119] Bin-Dahman O.A., Saleh T.A. (2020). Synthesis of Carbon Nanotubes Grafted with PEG and Its Efficiency for the Removal of Phenol from Industrial Wastewater. Environ. Nanotechnol. Monit. Manag..

[120] Cavallaro G., Lazzara G., Milioto S. (2011). Dispersions of Nanoclays of Different Shapes into Aqueous and Solid Biopolymeric Matrices. Extended Physicochemical Study. Langmuir.

[121] Taguet A., Cassagnau P., Lopez-Cuesta J.M. (2014). Structuration, Selective Dispersion and Compatibilizing Effect of (Nano)Fillers in Polymer Blends. Prog. Polym. Sci..

[122] Lu X., Huang J., Yang L., Zhang N., Jin G., Qu J. (2014). In-Situ Thermal Reduction and Effective Reinforcement of Graphene Nanosheet/Poly (Ethylene Glycol)/Poly (Lactic Acid) Nanocomposites. Polym. Adv. Technol..

[123] Tripathi S.N., Saini P., Gupta D., Choudhary V. (2013). Electrical and Mechanical Properties of PMMA/Reduced Graphene Oxide Nanocomposites Prepared via in Situ Polymerization. J. Mater. Sci..

[124] Sun Y., Zheng H., Tan M., Wang Y., Tang X., Feng L., Xiang X. (2014). Synthesis and Characterization of Composite Flocculant PAFS-CPAM for the Treatment of Textile Dye Wastewater. J. Appl. Polym. Sci..

[125] Foladori P., Andreottola G., Ziglio G. (2015). Sludge Reduction Technologies in Wastewater Treatment Plants.

[126] Rajeswari A., Amalraj A., Pius A. (2016). Adsorption Studies for the Removal of Nitrate Using Chitosan/PEG and Chitosan/PVA Polymer Composites. J. Water Process Eng..

[127] Perez-Calderon J., Marin-Silva D.A., Zaritzky N., Pinotti A. (2023). Eco-Friendly PVA-Chitosan Adsorbent Films for the Removal of Azo Dye Acid Orange 7: Physical Cross-Linking, Adsorption Process, and Reuse of the Material. Adv. Ind. Eng. Polym. Res..

[128] USEPA EPA (2002). Water Quality Standards Handbook.

[129] Jeong H., Kim H., Jang T. (2016). Irrigation Water Quality Standards for Indirect Wastewater Reuse in Agriculture: A Contribution toward Sustainablewastewater Reuse in South Korea. Water.

[130] Tiwari P. (2005). Proximate Analysis. Chem. Anal..

[131] (1986). Indian Standard Guidlines for Irrigation Water.

[132] Department of Water Affairs and Forestry (1996). Water Quality Guidelines. Agricultural Use: Irrigation Department of Water Affairs and Forestry.

